# Molecular classification of hormone receptor-positive /HER2-positive breast cancer reveals potential neoadjuvant therapeutic strategies

**DOI:** 10.1038/s41392-025-02181-3

**Published:** 2025-03-26

**Authors:** Chao Liu, Lisha Sun, Nan Niu, Pengjie Hou, Guanglei Chen, Hao Wang, Zhan Zhang, Xiaofan Jiang, Qianshi Xu, Yafei Zhao, Yimin Wang, Yuan Shi, Mingxin Liu, Yongliang Yang, Wei Qian, Jiandong Wang, Caigang Liu

**Affiliations:** 1 Cancer Stem Cell and Translation Medicine Lab, Shengjing Hospital of China Medical University, Shenyang, China; 2 Department of Oncology, Shengjing Hospital of China Medical University, Shenyang, China; 3 Department of Breast Surgery, Anyang Tumor Hospital, The Affiliated Anyang Tumor Hospital of Henan University of Science and Technology, Anyang, China; 4 Shanghai General Medical Center, School of Clinical Medicine, Shanghai University of Medicine & Health Science, Shanghai, China; 5 College of Medicine and Biological Information Engineering, Northeastern University, Shenyang, China; 6 Department of General Surgery, The First Medical Center of Chinese PLA General Hospital, Beijing, China

**Keywords:** Breast cancer, Breast cancer

## Abstract

Significant heterogeneity exists in hormone receptor (HR)-positive/HER2-positive (HR^+^/HER2^+^) breast cancer, contributing to suboptimal pathological complete response rates with conventional neoadjuvant treatment regimens. Overcoming this challenge requires precise molecular classification, which is pivotal for the development of targeted therapies. We conducted molecular typing on a cohort of 211 patients with HR^+^/HER2^+^ breast cancer and performed a comprehensive analysis of the efficacy of various neoadjuvant treatment regimens. Our findings revealed four distinct molecular subtypes, each exhibiting unique characteristics and therapeutic implications. The HER2-enriched subtype, marked by activation of the HER2 signaling and hypoxia-inducible factor 1 (HIF-1) pathway, may benefit from intensified anti-HER2-targeted therapy. Estrogen receptor (ER)-activated subtype demonstrated potential sensitivity to combined therapeutic strategies targeting both ER and HER2 pathways. Characterized by high immune cell infiltration, the immunomodulatory subtype showed sensitivity to HER2-targeted antibody–drug conjugates (ADCs) and promise for immune checkpoint therapy. The highly heterogeneous subtype requires a multifaceted therapeutic approach. Organoid susceptibility assays suggested phosphoinositide 3-kinase inhibitors may be a potential treatment option. These findings underscore the importance of molecular subtyping in HR^+^/HER2^+^ breast cancer, offering a framework for developing precise and personalized treatment strategies. By addressing the heterogeneity of the disease, these approaches have the potential to optimize therapeutic outcomes and improve patient care.

## Introduction

Approximately 70% of HER2-positive breast cancers are classified as hormone receptor-positive/HER2-positive (HR^+^/HER2^+^), a subtype with distinct clinicopathological features and a unique biological profile driven by the dual activation of estrogen receptor (ER) and HER2 signaling pathways.^1–4^ Although standard chemotherapy combined with HER2-targeted therapies, such as trastuzumab and pertuzumab, has become the cornerstone of treatment for HER2-positive breast cancer, the clinical efficacy of these regimens in HR^+^/HER2^+^ breast cancer is notably less favorable compared to HR^−^/HER2^+^ cases.^5–10^ This discrepancy highlights the biological differences between these subgroups and suggests that HR^+^/HER2^+^ breast cancer represents a distinct clinical subtype, with specific therapeutic challenges that remain unmet. A deeper understanding of this subtype is essential for improving clinical outcomes. Notably, gene expression profiling has revealed that ~30% of HR^+^/HER2^+^ breast cancers, as identified by the PAM50 assay, are classified as the HER2-enriched (HER2-E) intrinsic subtype.^11,12^ This subtype is characterized by robust activation of the ERBB2 pathway, coupled with relative suppression of ER signaling, correlating with enhanced sensitivity to HER2-targeted therapies such as trastuzumab, pertuzumab, and tyrosine kinase inhibitors (TKIs) like lapatinib and pyrotinib. In contrast, the majority (~70%) of HR^+^/HER2^+^ breast cancers exhibit a more luminal-like phenotype.^11,12^ For these patients, the efficacy of standard anti-HER2 and chemotherapy therapies remain limited, reflecting a critical unmet clinical need and driving the search for novel therapeutic strategies that can more effectively target both the ER and HER2 signaling axes while addressing the mechanisms of primary and acquired resistance.

HR^+^/HER2^+^ breast cancers exhibit considerable biological heterogeneity, driven by the intricate and dynamic crosstalk between ER and HER2 signaling pathways, which represents a crucial area of research.^13–16^ This complicated signaling interaction not only contributes to the oncogenic potential of HR^+^/HER2^+^ tumors but also plays a central role in resistance to HER2-targeted therapies, posing significant challenges in clinical management. Emerging evidence suggests that dual or multi-pathway blockade—targeting ER, HER2, and key downstream effectors such as cyclin-dependent kinase 4/6 (CDK4/6) and phosphoinositide 3-kinase (PI3K)—may offer a promising strategy to overcome this resistance and improve treatment outcomes.^3,14,17–21^ Preclinical and clinical studies have demonstrated that combining endocrine therapy with HER2-targeted agents and cell cycle inhibitors produces synergistic antitumor activity. These findings indicate that a rational multi-pathway inhibition strategy may offer a more efficacious approach for treating HR^+^/HER2^+^ breast cancer, potentially leading to improved clinical outcomes in this therapeutically challenging subgroup.^14,15,18,20,22^ Our comprehensive molecular analysis identified a synergistic mechanism for such multiple-pathway targeted blockade, providing a strong scientific rationale for the development of novel combination regimens that are specifically tailored to the unique biology of HR^+^/HER2^+^ disease.^23^

The MUKDEN 01 clinical trial represented a significant step in this direction, evaluating the efficacy of a new therapeutic strategy that concurrently inhibits ER, HER2, and cell cycle signaling pathways in HR^+^/HER2^+^ breast cancer. The trial combined an aromatase inhibitor (letrozole), a CDK4/6 inhibitor (dalpiciclib), and a HER2-targeted TKI (pyrotinib), achieving a pathologic complete response (pCR) rate of 30.4%. This promising outcome was comparable to the results achieved with standard HER2-targeted therapy combined with chemotherapy, while significantly reducing adverse effects and improving patients’ quality of life.^24^ These findings highlight the potential of chemotherapy-free regimen based on multi-pathway inhibition as a viable and more tolerable alternative for a substantial subset of patients with HR^+^/HER2^+^ breast cancer. However, despite these advances, challenges persist due to the inherent biological heterogeneity within HR^+^/HER2^+^ tumors. This variability within HR^+^/HER2^+^ breast cancer leads to differential responses to various treatment regimens, making it difficult to identify the optimal therapeutic approach for individual patients. Thus, there is a critical need to investigate the intrinsic characteristics of HR^+^/HER2^+^ breast cancer and define precise molecular subtypes to guide personalized treatment selection.

To address this urgent clinical need, we conducted a series of neoadjuvant clinical trials (the MUKDEN Trials), designed to explore novel treatment strategies and deepen our understanding of the molecular pathogenesis of HR^+^/HER2^+^ breast cancer. These trials investigated various combinations of therapies, including letrozole, dalpiciclib, and pyrotinib with or without trastuzumab; anti-HER2 antibody–drug conjugate (ADC) combined with pyrotinib; and chemotherapy, trastuzumab with or without pertuzumab. In parallel with these clinical investigations, we integrated transcriptomic data from pretreatment biopsy samples of patients who received these novel treatments, enabling a comprehensive molecular characterization of HR^+^/HER2^+^ breast cancer. Through unsupervised clustering and comprehensive gene expression profiling, we identified four distinct molecular subtypes of HR^+^/HER2^+^ tumors, each exhibiting unique biological signatures, pathway activation patterns, and differential treatment responses. To ensure the robustness, clinical relevance, and generalizability of these findings, we validated the four subtypes in an independent internal cohort as well as in three large, diverse public cohorts. Recognizing the need for a practical, cost-effective method for clinical application, we developed an immunohistochemistry (IHC)-based typing assay to approximate the mRNA-based classifications. Rigorous testing revealed a high level of concordance between IHC-based and mRNA-based classifications. This work has the potential to guide personalized therapeutic strategies for HR^+^/HER2^+^ breast cancer patients, optimizing outcomes by tailoring treatments to each tumor’s molecular profile.

## Results

### Study design and cohort information

**Fig. 1:**
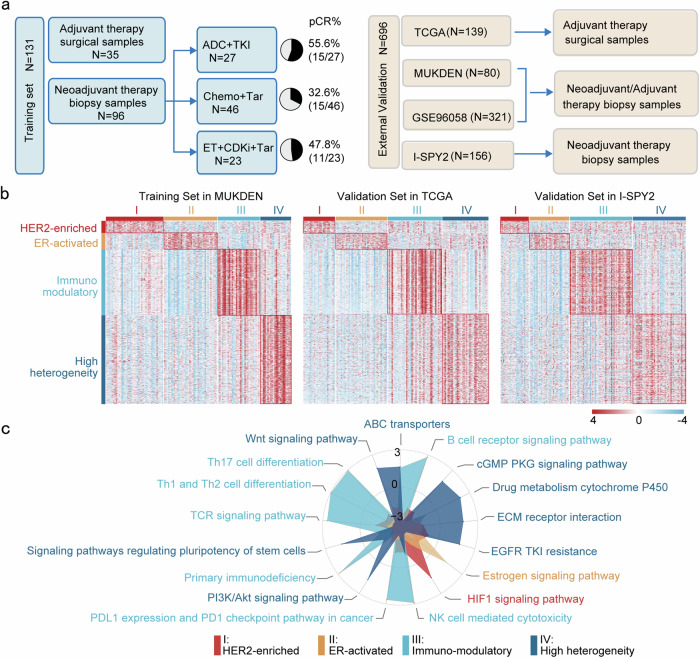
The mRNA subtypes and their association with clinical characteristics in the hormone receptor (HR)^+^/HER2^+^ breast cancer cohort. **a** The transcriptome cohort of HR^+^/HER2^+^ breast cancer patients. Chemo + Tar (chemotherapy plus anti-HER2-targeted therapy), ET + CDKi + Tar (endocrine therapy, CDK4/6 inhibitor, and anti-HER2-targeted therapy), ADC + TKI (anti-HER2 antibody–drug conjugate combined with tyrosine kinase inhibitor). **b** Heatmap depicting the mRNA expression profiles of the four subtypes in 131 samples and validation heatmap of 139 HR^+^/HER2^+^ breast cancer samples in The Cancer Genome Atlas Program (TCGA) and 156 HR^+^/HER2^+^ breast cancer samples in GEO (I-SPY2 trial). **c** Radar plot representing normalized enrichment score (NES) of each pathway feature in the corresponding cluster

We included 280 Chinese female patients diagnosed with HR^+^/HER2^+^ breast cancer who were treated at Shengjing Hospital of China Medical University and the People’s Liberation Army Hospital of China. After excluding cases where primary tumor tissue was unavailable or did not meet quality criteria, a total of 211 patients were included in the final analysis. Among them, 131 samples were assigned to the training set, while 80 samples were used for the independent validation set. Among the 131 patients, 96 received neoadjuvant therapy. The treatment regimens included ADC + TKI therapy (anti-HER2 ADC combined with TKI; *n* = 27, 20.6%), Chemo + Tar therapy (chemotherapy plus anti-HER2-targeted therapy; *n* = 46, 35.1%), and ET + CDKi + Tar therapy (endocrine therapy, CDK4/6 inhibitor, and anti-HER2-targeted therapy; *n* = 23, 17.6%). An additional 35 patients (26.7%) who received adjuvant therapy were classified as “Adjuvant” (Fig. 1a). Detailed drug administration protocols are provided in the “Materials and Methods” section. pCR was used as the efficacy measure, with pCR rates of 55.6%, 32.6%, and 47.8% observed in the ADC + TKI, Chemo + Tar, and ET + CDKi + Tar groups, respectively. Baseline clinicopathological characteristics for all patients are summarized in Supplementary Tables 1 and 2.

### Distinct biological characteristics among the four MUKDEN subtypes

Through non-negative matrix factorization (NMF) cluster analysis on 131 training samples, we identified four molecular subtypes for HR^+^/HER2^+^ breast cancer: MUKDEN I (*n* = 41), MUKDEN II (*n* = 38), MUKDEN III (*n* = 30), and MUKDEN IV (*n* = 22)—each with distinct gene expression profiles (Fig. 1b and Supplementary Fig. 1a).

Significant gene expression differences were determined using the adjusted *p* value < 0.05 and log2 fold change (FC) ≥ 1. A total of 1073 subtype-specific genes were identified, with 70, 95, 388, and 520 genes uniquely expressed in the MUKDEN I, II, III, and IV subtypes, respectively (Supplementary Table 3).

Subtype predictions were validated in an independent cohort (*n* = 80) and three large public datasets, including The Cancer Genome Atlas Program (TCGA)—BRCA cohort (*n* = 139), I-SPY2 trial cohort (*n* = 156), and GSE96058 cohort (*n* = 321). Submap analysis revealed strong correlations between the MUKDEN subtypes in the training set and their counterparts in these validation datasets, further demonstrating the robustness of the classification across diverse populations (Fig. 1b and Supplementary Fig. 1b).

Functional enrichment analysis using the Kyoto Encyclopedia of Genes and Genomes (KEGG) pathways revealed distinct biological characteristics associated with each subtype. We applied the same enrichment analysis method to the four validation datasets and found that the molecular features of the MUKDEN subtypes were consistently retained across these external datasets, as inferred from the classifier (Supplementary Figs. 2a–c and 3a, b).

We conducted gene set enrichment analysis (GSEA) on all genes in the training set to identify pathways associated with each subtype. In the MUKDEN I subtype, the HER2-related factors and the hypoxia-inducible factor 1 (HIF-1) signaling pathway were specifically upregulated, indicating a hypoxic tumor microenvironment (TME). The MUKDEN II subtype exhibited activation of the ER pathway, while the MUKDEN III subtype showed elevated levels of immune-related pathways compared to the other subtypes, suggesting an immunoregulatory phenotype. The MUKDEN IV subtype demonstrated a high degree of heterogeneity, with upregulation of pathways associated with poor prognosis, including extracellular matrix (ECM), PI3K–AKT, Wnt, EGFR, and drug metabolism-cytochrome P450 signaling pathways (Fig. 1c).

### Characterization of MUKDEN subtypes and neoadjuvant therapy

**Fig. 2:**
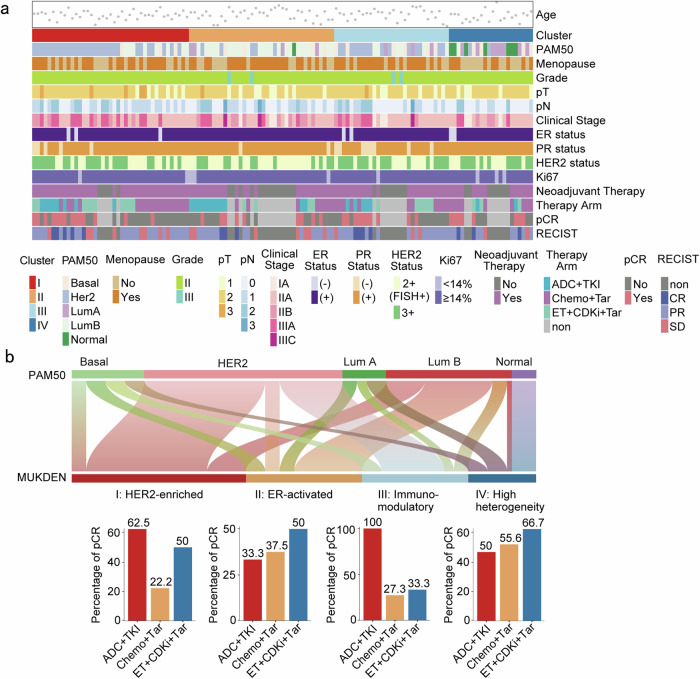
Clinical and treatment information for MUKDEN subtypes. **a** Association of the four subtypes with clinical characteristics, including pathological grade, PAM50 subtype, estrogen receptor (ER) status, progesterone receptor (PR) status, HER2 status, response evaluation criteria in solid tumors (RECIST), and pathologic complete response (pCR). **b** Sankey plot between PAM50 subtypes (upper) and MUKDEN subtypes (lower), and bar graph of pCR rates for each MUKDEN subtype

Among the four MUKDEN subtypes, no significant differences were observed in patient age, pathological grade, or clinical stage at baseline (Fig. 2a, Supplementary Fig. 4a, Supplementary Table 4). The Sankey plot in Fig. 2b illustrates the transition of PAM50 subtypes into MUKDEN subtypes. Additionally, the bar graph of MUKDEN subtypes illustrates pCR rates by treatment group within each subtype (Fig. 2b). We subdivided the independent validation set, comprising 80 samples into subtypes and analyzed the neoadjuvant treatment regimens and efficacy of 44 of these patients, drawing conclusions consistent with the training set (Supplementary Fig. 4b).

A primary objective of the MUKDEN molecular classification was to enhance the percentage of patients achieving pCR and maximize the likelihood of achieving pCR. Specifically, among patients who received neoadjuvant therapies, 36 were classified as MUKDEN I, 24 as MUKDEN II, 22 as MUKDEN III, and 14 as MUKDEN IV. Based on the MUKDEN subtype classification, selecting the optimal treatment approach for each subtype could increase the average pCR rate to 68.5% (Fig. 2b and Supplementary Table 1).

### Characteristics of patients achieving pCR with clinical trial treatment regimens

To better understand the factors influencing sensitivity to different treatments, we compared the characteristics of the sensitive versus non-sensitive groups within the ADC + TKI, Chemo + Tar, and ET + CDKi + Tar subgroups (Supplementary Fig. 5a–c). We aimed to identify the pathways associated with sensitivity to ADC + TKI and ET + CDKi + Tar therapies and to define cohort features that respond to these novel treatment regimens. We found that the T-cell receptor (TCR), B-cell receptor (BCR), PD-L1, and HIF-1 signaling pathways were significantly enriched in the ADC + TKI sensitivity group, whereas the ER signaling pathway was predominantly enriched in the ET + CDKi + Tar sensitivity group (Supplementary Fig. 5d, e). These findings were consistent with our analysis of the four MUKDEN subtypes and the therapeutic outcomes associated with the different neoadjuvant regimens for each subtype.

### Enrichment of *ERBB2* and upregulation of HIF-1 signaling pathway in MUKDEN I subtype

**Fig. 3:**
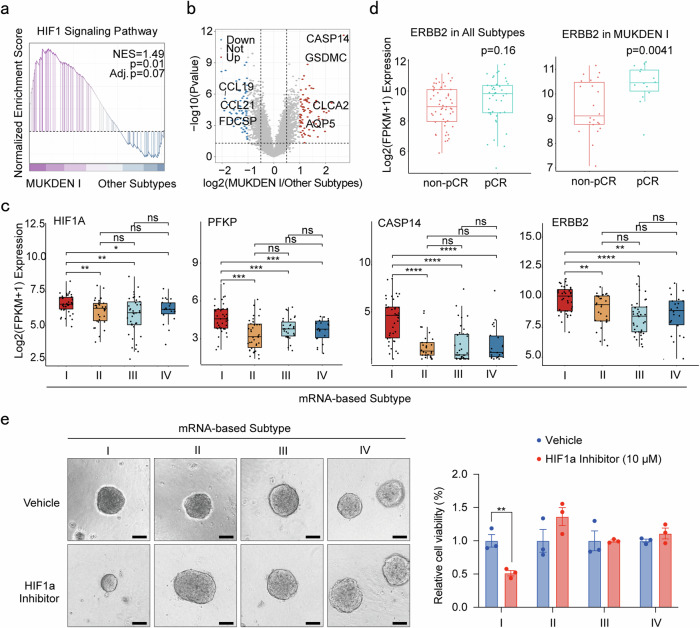
Activated HIF-1 signaling pathway and *ERBB2* factor in MUKDEN I subtype. **a** The gene set enrichment analysis (GSEA) enrichment analysis of HIF-1 signaling pathway in MUKDEN I subtype cohort. **b** Volcano plot of differential genes between MUKDEN I and other subtypes. **c** Boxplot showing the expression levels of HIF-1 signaling pathway genes (*ERBB2*, *CASP14*, *PFKP*, and *HIF1A*) among four subtypes. Boxplots show median (central line), upper and lower quartiles (box limits), 1.5× interquartile range (whiskers). *p < 0.05, **p < 0.01, ***p < 0.001, ****p < 0.0001; ns, no significance. **d** Expression of *ERBB2* in pCR and non-pCR patients for all subtypes (left) and MUKDEN I subtype (right). **e** Results of the cell viability assay testing the efficacy of HIF-1 inhibitor on patient-derived organoids (PDOs) from different MUKDEN subtypes. Three technical replicates were performed for each condition to ensure consistency and reproducibility of results. Scale bar: 200 μm

The HIF-1 signaling pathway was upregulated in the MUKDEN I subtype compared to the other subtypes. HER2-related genes and oxygen-deplete related genes (*HIF1A*, *PFKP, CASP14*, and *ERBB2*) were exclusively upregulated in this subtype (Fig. 3a–c and Supplementary Fig. 6a, b). Following the initiation of anti-HER2 therapy in study participants, we investigated the potential correlation between *ERBB2* expression and response to anti-HER2-targeted therapies. Divergence in treatment response was observed between the pCR and non-pCR groups, specifically within the MUKDEN I subtype, while no significant differences were found in the overall population or in the other subtypes. These results suggest a potential association between *ERBB2* expression levels and treatment sensitivity in the MUKDEN I subtype (Fig. 3d). Additionally, the HIF-1 signaling pathway was upregulated in the MUKDEN I subtype, and inhibition of this pathway using an HIF-1 signaling inhibitor suppressed the proliferation of patient-derived organoids (PDOs) from MUKDEN I tumors. These findings suggest that targeting the HIF-1 signaling pathway may offer a promising therapeutic approach for this subtype (Fig. 3e).

### Increased expression of estrogen-related genes in MUKDEN II subtype

**Fig. 4:**
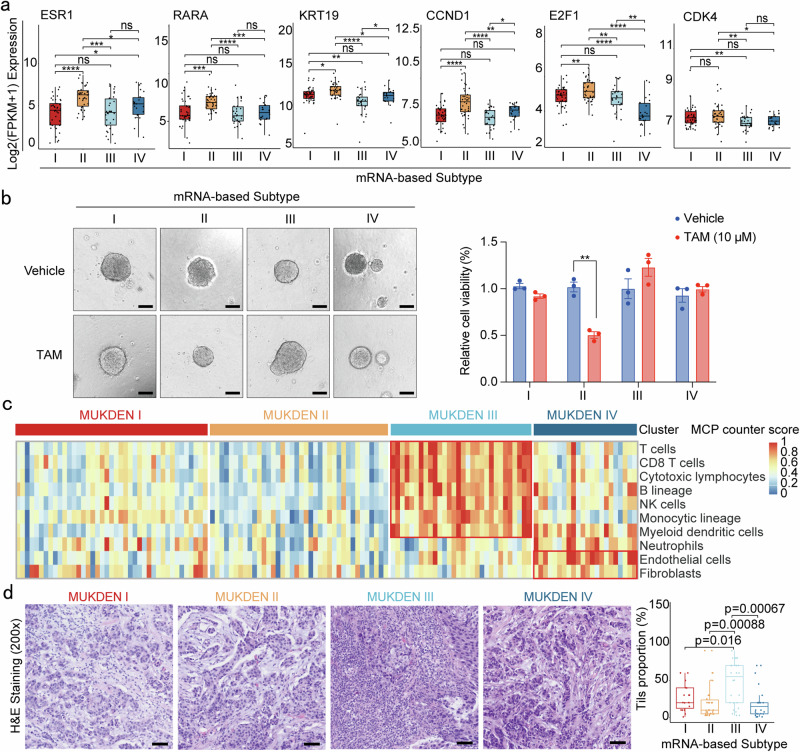
Microenvironment landscape and molecular characterization of MUKDEN II and III subtype. **a** Boxplot showing the expression levels of ER and cell cycle signaling pathway genes among four subtypes. Boxplots show median (central line), upper and lower quartiles (box limits), 1.5× interquartile range (whiskers). *p < 0.05, **p < 0.01, ***p < 0.001, ****p < 0.0001; ns, no significance. **b** Results of the cell viability assay testing the efficacy of tamoxifen (TAM) on PDOs from different MUKDEN subtypes. Three technical replicates were performed for each condition to ensure consistency and reproducibility of results. Scale bar: 200 μm. **c** Heatmap showing the estimated abundance of 10 microenvironmental cell types (MCP-Counter) among the four MUKDEN subtypes. The color represents the expression of the microenvironment cell, high expression is shown in red, while low expression is shown in blue. **d** Tumor-infiltrating lymphocyte levels in the four MUKDEN subtypes. Scale bar: 100 μm

The MUKDEN II subtype is characterized by elevated expression of ER and cell cycle-related genes, such as *ESR1* (estrogen receptor 1) and *CCND1* (cyclin D1) (Fig. 4a and Supplementary Fig. 7a–c). Notably, we observed that the pCR rate for the ADC + TKI treatment was relatively low (3/11) in the MUKDEN II subtype. GSEA and KEGG revealed downregulation of immune-related pathways in this subtype (Fig. 1c and Supplementary Figs. 2, 3). In contrast, the pCR rate for the ET + CDKi + Tar treatment was higher (2/4) in the MUKDEN II subtype. The observed upregulation of the ER pathway in MUKDEN II (Supplementary Fig. 5d), combined with the ability of ET + CDKi + Tar therapy to simultaneously block both ER and HER2 signaling, may contribute to the improved therapeutic efficacy seen in this subtype. Furthermore, we found that tamoxifen (TAM) effectively inhibited the growth of PDOs from MUKDEN II tumors (Fig. 4b), reinforcing the potential role of ER pathway modulation in the treatment of this subtype.

### Immunomodulation in the MUKDEN III subtype

The TME plays a pivotal role in tumor formation and progression. To gain a deeper understanding of the microenvironmental characteristics across the four MUKDEN subtypes, we conducted a comprehensive analysis. MUKDEN III tumors exhibited a significant increase in immune cell populations, as quantified by the Microenvironmental Cell Population Counter (MCP-counter)^25,26^ (Fig. 4c and Supplementary Figs. 8a–d, 9a, b).

Among the four subtypes, MUKDEN III exhibited significant differences in ten distinct immune cell populations. Moreover, this subtype was characterized by increased expression of five potentially targetable immune checkpoint molecules compared to the other subtypes (Supplementary Fig. 8e). A higher density of tumor-infiltrating lymphocytes (TILs) was observed in MUKDEN III tumors on hematoxylin and eosin (H&E)-stained sections (Fig. 4d).

Currently, the standard neoadjuvant treatment for HR^+^/HER2^+^ breast cancer does not incorporate immunotherapy. In our treatment subgroup analysis, patients with MUKDEN III tumors treated with the ET + CDKi + Tar regimen exhibited a lower pCR rate. In contrast, nearly all patients treated with ADC + TKI achieved a pCR (Fig. 2b). According to the GSEA, immune-related pathways were highly activated in MUKDEN III, while estrogen response pathways were suppressed (Fig. 1c). The pCR rate in the ADC + TKI treatment group increased in this subtype, which showed immune-related pathways upregulation according to the GSEA pathway enrichment analysis (Supplementary Fig. 5e). These findings suggest that the observed effects may result from mechanisms such as antibody-dependent cell-mediated cytotoxicity, complement-dependent cytotoxicity, and the cytotoxic effects of ADCs. Given this immune activation profile, ADC + TKI appears to be a promising therapeutic strategy for MUKDEN III. Additionally, immunosuppressive therapies targeting elevated immune checkpoint factors could represent another viable strategy. Further studies are warranted to investigate these therapeutic strategies in patients with this unique subtype.

### High heterogeneity in the MUKDEN IV subtype

**Fig. 5:**
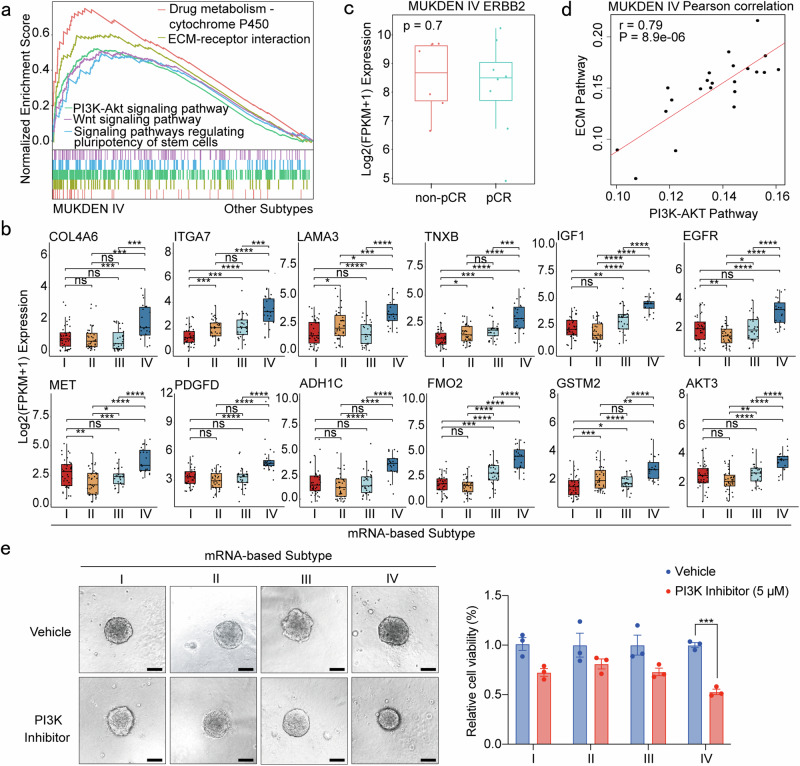
Highly heterogeneous characteristics of MUKDEN IV subtype. **a** The GSEA enrichment analysis of major feature signaling pathways in MUKDEN IV subtype cohort. **b** Boxplot showing the expression levels of the PI3K–AKT, ECM, Wnt, and EGFR signaling pathways genes among four subtypes. Boxplots show median (central line), upper and lower quartiles (box limits), 1.5× interquartile range (whiskers). *p < 0.05, **p < 0.01, ***p < 0.001, ****p < 0.0001; ns, no significance. **c** Boxplot showing the *ERBB2* expression in pCR and non-pCR patients of MUKDEN IV subtype. **d** Correlation between the PI3K–AKT and ECM signaling pathways in the MUKDEN IV subtype. The enrichment score for each sample in each gene set was calculated using the single-sample GSEA (ssGSEA) algorithm. **e** Results of the cell viability assay testing the efficacy of PI3K inhibitor on PDOs from different MUKDEN subtypes. Three technical replicates were performed for each condition to ensure consistency and reproducibility of results. Scale bar: 200 μm

The MUKDEN IV subtype, characterized by the upregulation of multiple aggressive signaling pathways and genes, was designated the “high-heterogeneity” subtype. This subtype exhibited enhanced activity in pathways associated with drug metabolism, such as ATP-binding cassette transporters, drug metabolism-cytochrome P450, and EGFR TKI resistance, distinguishing it from the other subtypes (Fig. 5a, b and Supplementary Fig. 10a, b).

Furthermore, the MUKDEN IV subtype exhibited significantly elevated expression of ECM-related genes, such as *IGF1*, *EGFR*, and *LAMA3*, most of which were associated with poor prognosis (Fig. 5a, b and Supplementary Fig. 10a, b). Within this subtype, increased expression of the Wnt signaling pathway was observed, along with other pathways associated with stem cell-like properties (Fig. 5a).

Significantly higher populations of stromal cells, particularly endothelial cells and fibroblasts, were identified in the MUKDEN IV subtype through MCP analysis. These findings were consistent with the enrichment of the ECM signaling pathway observed in this subtype (Fig. 4c and Supplementary Fig. 8a–d, 9b).

Our study also revealed the upregulation of the PI3K–AKT signaling pathway in the MUKDEN IV subtype. However, no significant differences in *ERBB2* expression were observed between the pCR and non-pCR groups within this subtype (Fig. 5c). Moreover, we found high enrichment of the PI3K–AKT signaling and ECM–receptor interaction pathways in the MUKDEN IV subtype. Subsequently, we performed a correlation analysis using single-sample GSEA (ssGSEA), which demonstrated a significant positive correlation between these two pathways (Fig. 5d and Supplementary Fig. 11a). Notably, inhibition of the PI3K–AKT signaling pathway significantly suppressed the proliferation of PDOs derived from MUKDEN IV tumors (Fig. 5e). These results provided a potential combination regimen for the treatment of this subtype.

### Immunohistochemical staining and classification of HR^+^/HER2^+^ of the four subtypes

To establish a practical IHC-based molecular classification system for HR^+^/HER2^+^ breast cancer, we systematically analyzed key genes associated with each of the four MUKDEN subtypes. Subsequently, genes enriched in characteristic signaling pathways within each subtype were described, reflecting the distinct biological features of each subtype. Candidate genes for IHC markers were selected based on their expression localization, extent of expression, and relevance, as identified in published literature and the Human Protein Atlas database. HIF-1 is the characteristic pathway in the HER2-enriched subtype, and the genes enriched in MUKDEN I include *ERBB2* and *PFKP*. Since *ERBB2* is not useful for distinguishing new subtypes, *PFKP* could serve as a MUKDEN I subtype marker through immunostaining.^27,28^ The ER pathway is characteristic of the endocrine-sensitive subtype, but it is unsuitable for distinguishing between subtypes. Analysis of expression localization data from published reports led to the selection of *GFRA1* as an immunohistochemical indicator for MUKDEN II subtype differentiation.^29–31^ *GFRA1* was selected from genes with a large expression difference in this subtype and a high correlation with *ESR1* factors. The immunoregulatory subtype was characterized by greater enrichment of immune-related genes than the other subtypes, with CD8 binding effector T cells. Previous studies have identified CD8A as an immunohistochemical marker of the immunoregulatory subtype.^32^ The highly heterogeneous subtype has complex gene expression involving multiple signaling pathways, making it difficult to represent all its characteristics with a single factor. We observed that the highly heterogeneous subtype exhibits rich tumor stroma characteristics. However, the commonly used clinical marker is KRT5 (Supplementary Fig. 12a). Thus, we demonstrated the transcriptional expression of *PFKP*, *GFRA1*, *CD8A*, and *KRT5* in MUKDEN subtypes (Supplementary Fig. 12b).

**Fig. 6:**
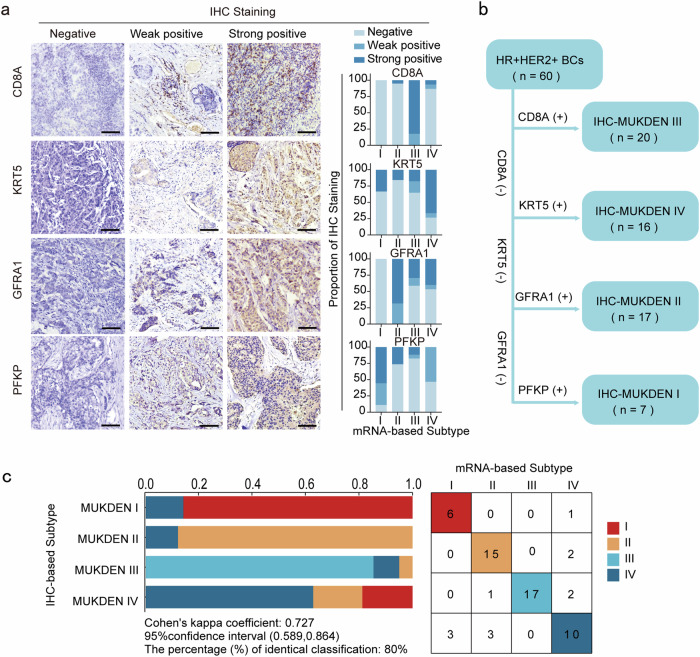
Analysis of agreement between immunohistochemistry (IHC)-based classification and mRNA-based classification. **a** Immunohistochemical staining criteria for the four factors *CD8A*, *KRT5*, *GFRA1*, *PFKP* (negative, weak positive, strong positive, scale bar 100 µm); the bar graph on the right represents the percentage of positivity for the factor in each subtype. **b** HR^+^/HER2^+^ breast cancer classification method based on IHC. **c** Agreement between IHC-based classification and mRNA-based classification for each subtype

Moreover, we demonstrated the transcriptional expression of *PFKP*, *GFRA1*, *CD8A*, and *KRT5* across the MUKDEN subtypes using TCGA datasets, revealing a correlation between mRNA and protein expression (Supplementary Fig. 12c). To further validate this correlation, immunohistochemical staining was performed to assess protein expression levels (Fig. 6a). Using a combination of these markers, 60 HR^+^/HER2^+^ breast cancer patients were classified into the following subtypes: (a) IHC-III: CD8A^+^; (b) IHC-IV: CD8A^−^ and KRT5^+^; (c) IHC-II: CD8A^−^, KRT5^−^ and GFRA1^+^; (d) IHC-I: CD8A^−^, KRT5^−^, GFRA1^−^ and PFKP^+^ (Fig. 6b). Cohen’s *κ* statistical analysis was used to assess the concordance between the IHC-based and mRNA-based classifications. The calculated Cohen’s *κ* coefficient was 0.727 (95% CI, 0.589–0.864), suggesting substantial agreement between the two methods (Fig. 6c). As shown in the confusion matrix, 80% of the samples were consistently classified using both methods (Fig. 6c).

## Discussion

Our transcriptomic analysis revealed significant diversity within HR^+^/HER2^+^ breast cancer, enabling the classification of these HR^+^/HER2^+^ populations into four distinct subtypes. The HER2-enriched subtype (MUKDEN I) was characterized by upregulated *ERBB2* expression and enhanced activity of the HIF-1 signaling pathway. The ER-activated subtype (MUKDEN II) was defined by the activation of the ER signaling pathway. The immunomodulatory subtype (MUKDEN III) exhibited increased immune cell infiltration and enhanced immune**-**modulatory activities. The highly heterogeneous subtype (MUKDEN IV) demonstrated significant variability in multifaceted regulation via numerous poor prognosis-related signaling pathways. Additionally, an IHC-based molecular classification system to facilitate the identification of these subtypes (Supplementary Fig. 13).

Clinical trials have reported lower pCR rates in patients with HR^+^/HER2^+^ breast cancer compared to those with HR^−^/HER2^+^ breast cancer.^5,6,20,33–35^ Due to the multi-target co-regulatory properties of HR^+^/HER2^+^ breast cancer, the inhibition of multiple signaling pathways has proven effective in preventing cross-resistance between therapies. This strategy is anticipated to enhance the efficacy of combination treatments. Moreover, the landscape of targeted therapies for breast cancer is rapidly evolving, with the introduction of innovative options, including anti-HER2-ADCs, anti-HER2-TKIs, endocrine therapies, CDK4/6 inhibitors, PI3K inhibitors, and immune checkpoint inhibitors (ICIs).^22,36–39^ The continuous development of novel drugs and combinational regimens has challenged existing efficacy prediction models, underscoring the need to optimize treatment strategies. At present, a precise classification for HR^+^/HER2^+^ breast cancer is lacking, emphasizing the urgent need to refine therapeutic approaches for this subtype.

Several clinical trials have investigated the potential of molecular profiling to guide neoadjuvant treatment strategies in breast cancer. The PerELISA and PAMELA studies, in particular, provided valuable insights by demonstrating that patients with the HER2-E subtype of HR^+^/HER2^+^ breast cancer, as classified by PAM50, exhibited significantly higher pCR rates compared to other subtypes. In these studies, pCR rates for the HER2-E subtype ranged from 41% to 54%, whereas other subtypes exhibited lower pCR rates, ranging from 13.8% to 28%.^40,41^ These findings underscore the importance of precise molecular classification in improving treatment outcomes. Additionally, the I-SPY trial expanded on this approach by integrating comprehensive datasets, including transcriptomic, proteomic, and clinical response data, to further refine breast cancer classification based on therapeutic response. By utilizing such detailed molecular profiling, these trials highlight the promise of tailoring treatment strategies to enhance efficacy, potentially transforming the landscape of neoadjuvant therapies in breast cancer.^42^

These studies, however, did have several limitations. Despite the extensive data on neoadjuvant therapy in the I-SPY2 study, the analytical methodology did not incorporate an unsupervised clustering model and exclusively categorized cancers with established treatment regimens. This approach limits its adaptability to novel therapeutic modalities. Furthermore, data on neoadjuvant endocrine therapy—an essential option for HR^+^/HER2^+^ breast cancer patients—was lacking, creating a gap in the comprehensive understanding of this treatment option. Although the PAM50 molecular classification has been instrumental in guiding treatment decisions across various breast cancer subtypes.^43,44^ It is limited in its ability to capture the full molecular heterogeneity of HR^+^/HER2^+^ breast cancer. Given the increasing array of targeted treatment options for individual patients, the PAM50 classification falls short in providing precise guidance for emerging targeted therapies.^45–47^

Our study provides a comprehensive analysis of the therapeutic potential for HR^+^/HER2^+^ breast cancer based on the molecular characteristics of the MUKDEN subtypes. The MUKDEN I subtype represents patients most likely to benefit from combination therapy involving multiple anti-HER2 agents. The intrinsic characteristics of the MUKDEN II subtype closely resemble those of traditional HR^+^/HER2^+^ breast cancer, demonstrating significant efficacy with combined ER and HER2 blockade. Moreover, the addition of CDK4/6 inhibitors further enhances treatment efficacy, consistent with prior reports.^23^ This subtype may particularly benefit from intensive endocrine therapy, emphasizing the value of combining hormonal and targeted therapies in this population. Our study revealed that the MUKDEN III subtype is characterized by elevated immune-related signaling pathways and TILs. Notably, this subtype exhibits increased mRNA expression of ICI genes, such as PD-L1 and CTLA-4, suggesting that ICIs have the potential to provide significant therapeutic benefits for these patients. The MUKDEN IV subtype suggests potential therapeutic strategies involving PI3K–AKT inhibitors and anti-stem cell therapies. While our approach is primarily focused on the neoadjuvant setting, it also holds promise for guiding personalized medicine in advanced therapeutic contexts.^48–50^

Although molecular typing of HR^+^/HER2^+^ breast cancer using transcriptome sequencing provides a comprehensive framework for understanding the tumor’s complex biology and developing targeted treatment strategies for each subtype, its widespread clinical application remains limited. The primary barriers to implementation are the significant time and financial costs associated with transcriptome sequencing, which hinder its routine use in clinical practice. To address these challenges, we have developed a clinically feasible IHC-based typing strategy. This approach simplifies the molecular classification of HR^+^/HER2^+^ breast cancer, enabling a more straightforward and cost-effective means of identifying distinct subtypes within HR^+^/HER2^+^ breast cancer. By utilizing IHC, a widely available and established technique in clinical settings, our method facilitates the translation of molecular insights into actionable clinical decisions. This strategy enhances the feasibility of personalized treatment regimens tailored to the unique molecular features of HR^+^/HER2^+^ breast cancer, offering promise for improved patient outcomes in daily clinical practice.

Our study had several limitations. First, although we utilized a well-annotated clinical dataset from neoadjuvant multi-arm targeted and chemotherapy molecular studies, the relatively small sample sizes within individual treatment arms and further stratification by subtypes have limited the statistical power. Second, multi-omics analyses are needed to further refine and validate the typing results, as they could offer a more integrated understanding of tumor biology. Third, the current findings were derived from retrospective data analysis. To confirm the current conclusions, prospective clinical trials are necessary. Lastly, we plan to conduct a comprehensive study that incorporates both classification and long-term follow-up to evaluate the survival benefits of our proposed approach. Additionally, further investigations are needed to validate the efficacy of novel therapeutic agents tailored to the MUKDEN subtypes.

In conclusion, our transcriptomic analysis of HR^+^/HER2^+^ breast cancer identified distinct molecular subtypes, highlighting the considerable biological heterogeneity within this population. This work has the potential to facilitate more personalized treatment strategies, ultimately improving clinical outcomes for patients with HR^+^/HER2^+^ breast cancer.

## Materials and methods

### Experimental models and object details

This study comprised five cohorts. The first cohort consisted of 131 patients with HR^+^/HER2^+^ breast cancer, all of whom were treated between January 1, 2020, and June 1, 2023, at the Cancer Centre of Shengjing Hospital of China Medical University and the People’s Liberation Army Hospital of China. RNA sequencing (RNA-seq) was performed on pretreatment biopsy samples from these patients. Detailed descriptions of the cohort, bioinformatics methods, and immunohistochemical analyses are provided in the Supplementary Information. The second cohort comprised 80 patients with HR^+^/HER2^+^ breast cancer who were treated between June 1, 2023, and September 1, 2024, at Shengjing Hospital of China Medical University. The third cohort comprised 139 patients with HR^+^/HER2^+^ breast cancer, identified from TCGA (http://cancergenome.nih.gov/). The fourth cohort included 156 patients with HR^+^/HER2^+^ breast cancer whose data were publicly available in the GEO database (GSE196096) (http://www.ncbi.nlm.nih.gov/geo/). The fifth cohort included 321 patients with HR^+^/HER2^+^ breast cancer whose data were publicly available in the GEO database (GSE96058) (http://www.ncbi.nlm.nih.gov/geo/). In our study, the first cohort was used for the molecular classification of HR^+^/HER2^+^ breast cancer, and the remaining cohorts were employed to validate the findings.

The first and second cohorts met the following inclusion criteria: (1) female patients with unilateral invasive ductal carcinoma who were positive for the ER or progesterone receptor (PR) phenotypes, as well as positive for HER2. (2) The pathology department of Shengjing Hospital conducted central pathological examination of tumor specimens. According to the guidelines of the American Society of Clinical Oncology/American Society of Pathologists, we used more than 10% (normal 1%, we actually chose 10%) positively stained cells as the cut-off value for ER/PR positive immunohistochemical detection. HER2 positivity was defined as either 3+ by IHC or 2+ with fluorescence in situ hybridization positivity. (3) No evidence of distant metastasis at diagnosis; patients with breast carcinoma in situ or inflammatory breast cancer were excluded. All HR^+^/HER2^+^ breast cancer patients in our study who received neoadjuvant therapy also underwent endocrine therapy as part of their postoperative adjuvant treatment, in accordance with established guidelines. All tissue samples included in this study were approved by the ethics committee of Shengjing Hospital of China Medical University and the People’s Liberation Army Hospital of China. Prior to the initiation of the study, informed consent was obtained from all individual participants included in the research.

According to the treatment profile, our cohort included the MUKDEN 01 and MUKDEN 09 clinical trial subcohort (ET + CDKi + pyrotinib maleate ± H, *n* = 23), MUKDEN06 clinical trial subcohort (ADC + pyrotinib maleate, *n* = 27), and Chemo + Tar treatment subcohort (Chemotherapy + Trastuzumab + Pertuzumab, *n* = 46). Detailed descriptions of the treatment regimens are provided in the Supplementary Materials.

Treatment response was evaluated using MRI, in accordance with the response evaluation criteria in solid tumors (RECIST). Tumor response was evaluated and classified as complete remission (CR), partial remission (PR), stable disease (SD), or progressive disease (PD). Several clinical parameters were collected, including age, tumor size, lymph node status, histological grade, ER, PR, HER2, and Ki67 status, treatment regimen, RECIST, and TNM stage—as defined in the eighth edition of the American Joint Cancer Staging Committee. The Miller–Payne grading system was used to assess the pathological response following neoadjuvant therapy. Tumor tissue samples for transcriptome sequencing were collected from the patients prior to treatment.

### RNA sequencing sample preparation and data generation

For each sample, the preprocessed sequence was compared with the human reference genome sequence using STAR software (2.5.3a). Additionally, the FASTQ files were then mapped to the human reference genome (Gencode GRCh38, hg38). Fragments Per Kilobase of exon model per million mapped fragments (FPKM) is a widely used quantitative expression index in double-ended transcriptome sequencing that considers both gene length and sequencing data quantity. Typically, an FPKM value ≥ 0.1 signifies gene expression. Prior to subsequent analysis, genes with FPKM values of 0 in > 30% of the samples were excluded. Batch effects were removed through the R packages sva (v3.46.0).

### HR^+^/HER2^+^ breast cancer subtypes generated by unsupervised clustering analysis and their biological function

The NMF algorithm (“NMF” function in R) was employed to determine the optimal number of clusters for the HR^+^/HER2^+^ breast cancer dataset and to assign samples to these clusters. The top 20% genes based on the MAD were selected for the clustering process. The optimal number of clusters was determined by identifying the *k* value at which the cophenetic correlation coefficient decreased in magnitude.

The signature genes of each subtype were defined as differentially expressed in the subtype compared with other subtypes with log2 FC ≥ 1 and adjusted *p* value < 0.05. KEGG pathway enrichment analysis was performed to determine the biological function of each subtype.

### Validation of the MUKDEN subtyping in other independent cohorts

Our classification was compared with the predictions of HR^+^/HER2^+^ breast cancer cohorts in TCGA/GEO cohorts using nearest template prediction analyses module from Gene Pattern.

Subsequently, subclass mapping (SubMap) analyses (available in Gene Pattern modules, https://cloud.genepattern.org/), which compared the mRNA expression profiles of independent patient cohorts, were used to determine whether the subclasses identified in the training and validation sets were correlated. The results of the SubMap analysis were visualized using the R package “pheatmap.”

### Public HR^+^/HER2^+^ breast cancer dataset

This study used the GSE196096 and GSE96058 datasets, which represent the largest microarray cohorts containing comprehensive neoadjuvant therapy information within published breast cancer expression profiles, in conjunction with TCGA-BRCA cohort. The R packages “GEOquery” and “TCGAbiolinks” were used to retrieve the clinical data from the GEO datasets and the TCGA-BRCA cohort, respectively.

### Breast cancer intrinsic subtype

The “genefu” package (v2.12.0) was used to determine the inherent subtypes of breast cancer in our samples.

### Differential genes and pathways in therapy subcohort

Differentially expressed genes in the therapy subcohort were identified as those with a log2 FC ≥ 1 and a *p* value < 0.05 in the pCR and non-pCR groups. Pathway enrichment analysis of the overexpressed genes was conducted using the KEGG database. Additionally, pathway enrichment analysis was performed using GSEA with the ClusterProfiler R package (v3.18.1) to assess the concentration of pathways in each transcriptome subtype. GSEA evaluates whether a predefined set of genes exhibits a statistically significant association with cumulative changes in gene expression related to a specific phenotype. The samples were categorized based on their pCR and non-pCR characteristics and subsequently subjected to GSEA. The Molecular Signature Database (MSigDB) was used to analyze the selected gene set (C2). The enrichment score (ES) in GSEA was computed by initially arranging the genes in descending order of their significance in pCR and non-pCR, followed by assessing the distribution of genes within each gene set in the sorted list.

### ssGSEA and gene set variation analysis (GSVA)

Based on the mRNA expression matrix, the ssGSEA method implemented in the GSVA R package (v1.34.0) was used to calculate the PI3K–AKT and ECM–receptor interaction signaling pathway scores using the gene set (C2.all.v7.4. symbols) obtained from the MSigDB.

### Estimation of the number of immune cells in tumor tissue

MCP-counter was used to evaluate the absolute abundance of eight immune and two non-immune stromal cell populations. The ssGSEA method was also used to estimate immune infiltration, which calculates an enrichment score that represents the extent to which genes within a particular set are up and downregulated. The GSVA R package was used to estimate six additional immune cell populations: regulatory T cells (Tregs), T helper cells 1 and 2, T helper cells 17, effective memory T cells, and central memory T cells. The ESTIMATE algorithm was used to calculate immune and stromal scores, which could reflect the enrichment of genes in the stroma and immune cells.

### IHC staining

IHC staining was conducted on formalin-fixed paraffin-embedded (FFPE) tumor specimens (4-μm thick) from all cohorts to assess the expression of PFKP, GFRA1, CD8A, and KRT5. FFPE tissue sections were de-paraffinized, rehydrated, and treated with hydrogen peroxide to block endogenous peroxidase activity. Antigen retrieval was conducted using citrate buffer (pH 6) at an elevated temperature and pressure. Subsequently, tissue sections were incubated with primary antibodies against PFKP (PTG, 13389-1-AP, 1:200), GFRA1 (Bioss, bs-0201R, 1:200), CD8A (CST, KHC0014 kit), and KRT5 (Bioss, bs-20824R, 1:200) overnight at 4 °C. The antigen-antibody interaction was detected through a 3,3′-diaminobenzidine (DAB) kit (GeneTech, GK600511), following the manufacturer’s protocol, and the sections were examined under a light microscope (Echo Revolve, CA, USA). The agreement between the mRNA-based classification and IHC-based classification was analyzed using Cohen’s *κ* statistic.

### Evaluation of TILs

TILs were assessed in H&E-stained sections from Shengjing Hospital of China Medical University, which belonged to the first cohort. According to the International TILs Working Group recommendations, we assessed TILs using lymphocytes in the stroma between tumor nests.

### In vitro viability assay of PDOs

We collected PDO samples from HR^+^/HER2^+^ breast cancer patients who had undergone surgical procedures at Shengjing Hospital between January 1, 2020 and June 1, 2023 (Supplementary Table 5). PDO was generated according to the methodology described in a prior study. Briefly, we performed enzymatic digestion of breast cancer tissues using the Tumor Dissociation Kit, human (Miltenyi, 130-095-929), after sectioning them into 1–3 mm^3^ sections. To digest the samples, gentleMACS™ Dissociators were used, and ~1 h of the 37 °C_h_TDK_3 program was used. In the following steps, primary cells were cultured in 24-well plates, and when they reached a diameter of 50 μm, organoids were harvested and seeded into 96-well plates (Corning, 3599). To detect the drugs using the sandwich method, a base layer of 50–100 μL of 50% Matrigel (Corning, 356231; Matrigel mixed with PBS at a 1:1 ratio) was applied to the bottom of the culture plates prior to seeding. After 1 day of incubation, each well received 100 μL of organoid medium as well as an upper layer, followed by drug testing. Based on the manufacturer’s instructions, organoid viability was monitored using the Living Cell-Fluo Organoid Vitality Assay Kit (bioGenous, E238004). The company served as the source of the compounds, including inhibitors of the HIF-1 signaling pathway (KC7F2, Selleck, 927822-86-4), 4-hydroxytamoxifen (4-hydroxytamoxifen, Selleck, 68047-06-3), and PI3Ki (HS173, Selleck, 1276110-06-5).

### Statistical analysis

Comparisons of ordered class variables and continuous variables were performed by Student’s *t*-test and Kruskal–Wallis test, while categorical variables were compared using Pearson’s chi-square test and Fisher’s exact test. Differences among groups were analyzed using one-way analysis of variance (ANOVA) with a post hoc Newman–Keuls test. A Benjamini–Hochberg adjustment was applied to multiple comparisons to reduce false discovery rates. All statistical tests were two-sided, and specific methods were annotated in addition to the *p* value in the text. An analysis of the correlation between mRNA-based classification and IHC-based classification was conducted using Cohen’s *κ* statistic. All analyses were performed using R Statistical Software (https://cran.r-project.org/).

### Software

R (https://www.R-project.org/) and the Bioconductor software package (https://www.bioconductor.org/) were used for data statistics and bioinformatics analysis. We used R version 4.2.2.

## Supplementary information

Supplementary_Materials_Methods

## Data Availability

The mRNA-seq data generated in this study have been deposited in the Genome Sequence Archive (GSA) database under accession code HRA007185 (https://ngdc.cncb.ac.cn/gsa-human/submit/hra/submit). Protein expression data measured via mass spectrometry were downloaded from the cBioPortal (http://www.cbioportal.org). The remaining data are available within the Article or Source Data file.

## Code availability

No unpublished codes were used in this study. Additionally, details regarding the already-published code that was used are provided in the “Materials and Methods” and Supplementary Methods sections.

## References

[1] Schettini, F. et al. HER2-enriched subtype and pathological complete response in HER2-positive breast cancer: a systematic review and meta-analysis. *Cancer Treat. Rev.* **84**, 101965 (2020). 32000054 10.1016/j.ctrv.2020.101965 PMC7230134

[2] Howlader, N. et al. US incidence of breast cancer subtypes defined by joint hormone receptor and HER2 status. *J. Natl. Cancer Inst.* **106**, dju055 (2014). 24777111 10.1093/jnci/dju055 PMC4580552

[3] Chan, A. et al. Neratinib after trastuzumab-based adjuvant therapy in patients with HER2-positive breast cancer (ExteNET): a multicentre, randomised, double-blind, placebo-controlled, phase 3 trial. *Lancet Oncol.* **17**, 367–377 (2016). 26874901 10.1016/S1470-2045(15)00551-3

[4] Gianni, L. et al. Efficacy and safety of neoadjuvant pertuzumab and trastuzumab in women with locally advanced, inflammatory, or early HER2-positive breast cancer (NeoSphere): a randomised multicentre, open-label, phase 2 trial. *Lancet Oncol.* **13**, 25–32 (2012). 22153890 10.1016/S1470-2045(11)70336-9

[5] Cortazar, P. et al. Pathological complete response and long-term clinical benefit in breast cancer: the CTNeoBC pooled analysis. *Lancet* **384**, 164–172 (2014). 24529560 10.1016/S0140-6736(13)62422-8

[6] Schettini, F. et al. Hormone receptor/human epidermal growth factor receptor 2-positive breast cancer: where we are now and where we are going. *Cancer Treat. Rev.* **46**, 20–26 (2016). 27057657 10.1016/j.ctrv.2016.03.012

[7] Gianni, L. et al. Neoadjuvant chemotherapy with trastuzumab followed by adjuvant trastuzumab versus neoadjuvant chemotherapy alone, in patients with HER2-positive locally advanced breast cancer (the NOAH trial): a randomised controlled superiority trial with a parallel HER2-negative cohort. *Lancet* **375**, 377–384 (2010). 20113825 10.1016/S0140-6736(09)61964-4

[8] von Minckwitz, G. et al. Capecitabine in addition to anthracycline- and taxane-based neoadjuvant treatment in patients with primary breast cancer: phase III GeparQuattro study. *J. Clin. Oncol.* **28**, 2015–2023 (2010). 20308671 10.1200/JCO.2009.23.8303

[9] Schneeweiss, A. et al. Pertuzumab plus trastuzumab in combination with standard neoadjuvant anthracycline-containing and anthracycline-free chemotherapy regimens in patients with HER2-positive early breast cancer: a randomized phase II cardiac safety study (TRYPHAENA). *Ann. Oncol.* **24**, 2278–2284 (2013). 23704196 10.1093/annonc/mdt182

[10] Denkert, C. et al. Tumour-infiltrating lymphocytes and prognosis in different subtypes of breast cancer: a pooled analysis of 3771 patients treated with neoadjuvant therapy. *Lancet Oncol.* **19**, 40–50 (2018). 29233559 10.1016/S1470-2045(17)30904-X

[11] Prat, A. et al. Molecular features and survival outcomes of the intrinsic subtypes within HER2-positive breast cancer. *J. Natl. Cancer Inst.* **106**, dju152 (2014). 25139534 10.1093/jnci/dju152 PMC4151853

[12] Cejalvo, J. M. et al. Intrinsic subtypes and gene expression profiles in primary and metastatic breast cancer. *Cancer Res.* **77**, 2213–2221 (2017). 28249905 10.1158/0008-5472.CAN-16-2717 PMC5822682

[13] Debien, V., de Azambuja, E. & Piccart-Gebhart, M. Optimizing treatment for HER2-positive HR-positive breast cancer. *Cancer Treat. Rev.* **115**, 102529 (2023). 36921556 10.1016/j.ctrv.2023.102529

[14] Goel, S. et al. Overcoming therapeutic resistance in HER2-positive breast cancers with CDK4/6 inhibitors. *Cancer Cell* **29**, 255–269 (2016). 26977878 10.1016/j.ccell.2016.02.006 PMC4794996

[15] Alqaisi, A. et al. Impact of estrogen receptor (ER) and human epidermal growth factor receptor-2 (HER2) co-expression on breast cancer disease characteristics: implications for tumor biology and research. *Breast Cancer Res. Treat.* **148**, 437–444 (2014). 25257728 10.1007/s10549-014-3145-x

[16] García Fernández, A. et al. Survival and clinicopathological characteristics of breast cancer patient according to different tumour subtypes as determined by hormone receptor and Her2 immunohistochemistry. A single institution survey spanning 1998 to 2010. *Breast* **21**, 366–373 (2012). 22487206 10.1016/j.breast.2012.03.004

[17] Croessmann, S. et al. Combined blockade of activating ERBB2 mutations and ER results in synthetic lethality of ER+/HER2 mutant breast cancer. *Clin. Cancer Res.* **25**, 277–289 (2019). 30314968 10.1158/1078-0432.CCR-18-1544 PMC6320312

[18] Rimawi, M. F. et al. TBCRC023: a randomized phase II neoadjuvant trial of lapatinib plus trastuzumab without chemotherapy for 12 versus 24 weeks in patients with HER2-positive breast cancer. *Clin. Cancer Res.* **26**, 821–827 (2020). 31662331 10.1158/1078-0432.CCR-19-0851

[19] Hua, X. et al. Trastuzumab plus endocrine therapy or chemotherapy as first-line treatment for patients with hormone receptor-positive and HER2-positive metastatic breast cancer (SYSUCC-002). *Clin. Cancer Res.* **28**, 637–645 (2022). 34810217 10.1158/1078-0432.CCR-21-3435 PMC9377763

[20] Gianni, L. et al. Neoadjuvant treatment with trastuzumab and pertuzumab plus palbociclib and fulvestrant in HER2-positive, ER-positive breast cancer (NA-PHER2): an exploratory, open-label, phase 2 study. *Lancet Oncol.* **19**, 249–256 (2018). 29326029 10.1016/S1470-2045(18)30001-9

[21] Vasan, N., Toska, E. & Scaltriti, M. Overview of the relevance of PI3K pathway in HR-positive breast cancer. *Ann. Oncol.* **30**, x3–x11 (2019). 31859348 10.1093/annonc/mdz281 PMC6923788

[22] Johnston, S. R. D. et al. Phase III, randomized study of dual human epidermal growth factor receptor 2 (HER2) blockade with lapatinib plus trastuzumab in combination with an aromatase inhibitor in postmenopausal women with HER2-positive, hormone receptor-positive metastatic breast cancer: updated results of ALTERNATIVE. *J. Clin. Oncol.* **39**, 79–89 (2021). 32822287 10.1200/JCO.20.01894

[23] Bu, J. et al. Dalpiciclib partially abrogates ER signaling activation induced by pyrotinib in HER2(+)HR(+) breast cancer. *Elife* **12**, e85246 (2023). 36602226 10.7554/eLife.85246 PMC9822241

[24] Niu, N. et al. A multicentre single arm phase 2 trial of neoadjuvant pyrotinib and letrozole plus dalpiciclib for triple-positive breast cancer. *Nat. Commun.* **13**, 7043 (2022). 36396665 10.1038/s41467-022-34838-w PMC9672048

[25] Becht, E. et al. Estimating the population abundance of tissue-infiltrating immune and stromal cell populations using gene expression. *Genome Biol.* **17**, 218–238 (2016). 27908289 10.1186/s13059-016-1113-y PMC5134277

[26] Newman, A. M. et al. Robust enumeration of cell subsets from tissue expression profiles. *Nat. Methods* **12**, 453–457 (2015). 25822800 10.1038/nmeth.3337 PMC4739640

[27] Chen, J. et al. PFKP alleviates glucose starvation-induced metabolic stress in lung cancer cells via AMPK-ACC2 dependent fatty acid oxidation. *Cell Discov.* **8**, 52–68 (2022). 35641476 10.1038/s41421-022-00406-1 PMC9156709

[28] Lu, T. J. et al. Phosphofructokinase platelet overexpression accelerated colorectal cancer cell growth and motility. *J. Cancer* **14**, 943–951 (2023). 37151384 10.7150/jca.82738 PMC10158518

[29] Morandi, A. et al. GDNF-RET signaling in ER-positive breast cancers is a key determinant of response and resistance to aromatase inhibitors. *Cancer Res.* **73**, 3783–3795 (2013). 23650283 10.1158/0008-5472.CAN-12-4265 PMC3686594

[30] Esseghir, S. et al. A role for glial cell derived neurotrophic factor induced expression by inflammatory cytokines and RET/GFR alpha 1 receptor up-regulation in breast cancer. *Cancer Res.* **67**, 11732–11741 (2007). 18089803 10.1158/0008-5472.CAN-07-2343

[31] Esseghir, S. et al. Identification of transmembrane proteins as potential prognostic markers and therapeutic targets in breast cancer by a screen for signal sequence encoding transcripts. *J. Pathol.* **210**, 420–430 (2006). 17054309 10.1002/path.2071

[32] Chen, D. S. & Mellman, I. Elements of cancer immunity and the cancer-immune set point. *Nature* **541**, 321–330 (2017). 28102259 10.1038/nature21349

[33] de Azambuja, E. et al. Lapatinib with trastuzumab for HER2-positive early breast cancer (NeoALTTO): survival outcomes of a randomised, open-label, multicentre, phase 3 trial and their association with pathological complete response. *Lancet Oncol.* **15**, 1137–1146 (2014). 25130998 10.1016/S1470-2045(14)70320-1

[34] Carey, L. A. et al. Molecular heterogeneity and response to neoadjuvant human epidermal growth factor receptor 2 targeting in CALGB 40601, a randomized phase III trial of paclitaxel plus trastuzumab with or without lapatinib. *J. Clin. Oncol.* **34**, 542–549 (2016). 26527775 10.1200/JCO.2015.62.1268 PMC4980567

[35] Guarneri, V. et al. Prospective biomarker analysis of the randomized CHER-LOB study evaluating the dual anti-HER2 treatment with trastuzumab and lapatinib plus chemotherapy as neoadjuvant therapy for HER2-positive breast cancer. *Oncologist* **20**, 1001–1010 (2015). 26245675 10.1634/theoncologist.2015-0138 PMC4571802

[36] Kaufman, B. et al. Trastuzumab plus anastrozole versus anastrozole alone for the treatment of postmenopausal women with human epidermal growth factor receptor 2-positive, hormone receptor-positive metastatic breast cancer: results from the randomized phase III TAnDEM study. *J. Clin. Oncol.* **27**, 5529–5537 (2009). 19786670 10.1200/JCO.2008.20.6847

[37] Johnston, S. et al. Lapatinib combined with letrozole versus letrozole and placebo as first-line therapy for postmenopausal hormone receptor-positive metastatic breast cancer. *J. Clin. Oncol.* **27**, 5538–5546 (2009). 19786658 10.1200/JCO.2009.23.3734

[38] Rimawi, M. et al. First-line trastuzumab plus an aromatase inhibitor, with or without pertuzumab, in human epidermal growth factor receptor 2-positive and hormone receptor-positive metastatic or locally advanced breast cancer (PERTAIN): a randomized, open-label phase II trial. *J. Clin. Oncol.* **36**, 2826–2835 (2018). 30106636 10.1200/JCO.2017.76.7863

[39] von Minckwitz, G. et al. Trastuzumab emtansine for residual invasive HER2-positive breast cancer. *N. Engl. J. Med.* **380**, 617–628 (2019). 30516102 10.1056/NEJMoa1814017

[40] Guarneri, V. et al. De-escalated therapy for HR+/HER2+ breast cancer patients with Ki67 response after 2-week letrozole: results of the PerELISA neoadjuvant study. *Ann. Oncol.* **30**, 921–926 (2019). 30778520 10.1093/annonc/mdz055 PMC6594455

[41] Llombart-Cussac, A. et al. HER2-enriched subtype as a predictor of pathological complete response following trastuzumab and lapatinib without chemotherapy in early-stage HER2-positive breast cancer (PAMELA): an open-label, single-group, multicentre, phase 2 trial. *Lancet Oncol.* **18**, 545–554 (2017). 28238593 10.1016/S1470-2045(17)30021-9

[42] Wolf, D. M. et al. Redefining breast cancer subtypes to guide treatment prioritization and maximize response: predictive biomarkers across 10 cancer therapies. *Cancer Cell* **40**, 609–623.e6 (2022). 35623341 10.1016/j.ccell.2022.05.005 PMC9426306

[43] Cancer Genome Atlas Network. Comprehensive molecular portraits of human breast tumours. *Nature* **490**, 61–70 (2012). 10.1038/nature11412 PMC3465532 23000897

[44] Waks, A. G. & Winer, E. P. Breast cancer treatment: a review. *JAMA* **321**, 288–300 (2019). 30667505 10.1001/jama.2018.19323

[45] Krug, K. et al. Proteogenomic landscape of breast cancer tumorigenesis and targeted therapy. *Cell* **183**, 1436–1456.e31 (2020). 33212010 10.1016/j.cell.2020.10.036 PMC8077737

[46] Ades, F. et al. Luminal B breast cancer: molecular characterization, clinical management, and future perspectives. *J. Clin. Oncol.* **32**, 2794–2803 (2014). 25049332 10.1200/JCO.2013.54.1870

[47] Mayer, E. L. et al. Palbociclib with adjuvant endocrine therapy in early breast cancer (PALLAS): interim analysis of a multicentre, open-label, randomised, phase 3 study. *Lancet Oncol.* **22**, 212–222 (2021). 33460574 10.1016/S1470-2045(20)30642-2

[48] Guarneri, V. et al. Survival after neoadjuvant therapy with trastuzumab-lapatinib and chemotherapy in patients with HER2-positive early breast cancer: a meta-analysis of randomized trials. *ESMO Open* **7**, 100433 (2022). 35276440 10.1016/j.esmoop.2022.100433 PMC8917305

[49] Broglio, K. R. et al. Association of pathologic complete response to neoadjuvant therapy in HER2-positive breast cancer with long-term outcomes: a meta-analysis. *JAMA Oncol.* **2**, 751–760 (2016). 26914222 10.1001/jamaoncol.2015.6113

[50] Curigliano, G. et al. Tucatinib versus placebo added to trastuzumab and capecitabine for patients with pretreated HER2+ metastatic breast cancer with and without brain metastases (HER2CLIMB): final overall survival analysis. *Ann. Oncol.* **33**, 321–329 (2022). 34954044 10.1016/j.annonc.2021.12.005

