# Supplementary material for: Molecular classification of hormone receptor-positive /HER2-positive breast cancer reveals potential neoadjuvant therapeutic strategies
Source: Signal Transduct Target Ther. 2025 Mar 26;10:97. doi: 10.1038/s41392-025-02181-3 (PMC11937365; doi:10.1038/s41392-025-02181-3)
Supplement: Supplementary file 1 — Supplementary_Materials_Methods [file 41392_2025_2181_MOESM1_ESM.docx]

Supplementary Materials for

Molecular classification of hormone receptor-positive HER2-positive breast cancer reveals potential neoadjuvant therapeutic strategies.

Chao Liu^1,2†^, Lisha Sun^1,2†^, Nan Niu^1,2†^, Pengjie Hou^1,2†^, Guanglei Chen^1,2†^, Hao Wang^1,2^, Zhan Zhang^1,2^, Xiaofan Jiang^1,2^, Qianshi Xu^1,2^, Yafei Zhao^1,2^, Yimin Wang^1,2^, Yuan Shi^3^, Mingxin Liu^1,2^, Yongliang Yang^4^, Wei Qian^5^, Jiandong Wang^6^*, Caigang Liu^1,2^*

†These authors contributed equally to this work.

*Corresponding author

Correspondence to:

Caigang Liu, MD, PhD

Email: [liucg@sj-hospital.org](mailto:xxxxx@xxxx.xxx)

**This PDF file includes:**

Materials and Methods

Figures. S1 to S13

Tables S1 to S5

**Materials and Methods**

**Treatment program details**

Patients were recruited from three multicenter clinical trials conducted by our center: MUKDEN 01, MUKDEN 06, and MUKDEN 09. The MUKDEN 01 treatment regimen was as follows: patients received neoadjuvant therapy over five 4-week cycles. Each cycle consisted of pyrotinib (anti-HER2 TKI, 320 mg) and letrozole (2.5 mg) once daily for 28 days and dalpiciclib (CDK4/6 inhibitor, 125 mg) once daily for 21 days, followed by a 7-day withdrawal period. Premenopausal or perimenopausal patients additionally received goserelin (3.6 mg) subcutaneously on day 1 of each cycle for five cycles. Surgery was performed 2–4 weeks after the final neoadjuvant therapy.

The treatment regimen for the test group in the MUKDEN 09 clinical trial was as follows: the MUKDEN 01 regimen supplemented with trastuzumab, with an initial dose of 8 mg/kg followed by maintenance dose of 6 mg/kg every 3 weeks.

The protocol for the MUKDEN 06 trial group was as follows: patients received neoadjuvant therapy in one cycle every 3 weeks for a total of six cycles. For patients in the trial group, each cycle consisted of pyrotinib (320 mg) once daily for 21 days and ARX788 (anti-HER2-ADC) at a dose of 1.5 mg/kg via intravenous infusion every 3 weeks.

The protocol for control patients in the MUKDEN 06 and MUKDEN 09 clinical trials was as follows: patients received neoadjuvant therapy every 3 weeks for a total of six cycles. The patients received docetaxel at 75 mg/m^2^ per central venous infusion, carboplatin at an AUC of six per infusion every 3 weeks, trastuzumab at 6 mg/kg every 3 weeks after the first 8 mg/kg dose, and pertuzumab at 420 mg every 3 weeks after the first 840 mg dose. The primary efficacy endpoint was pathologic complete response (pCR).

Tumor response was evaluated using MRI scans and categorized per the Response Evaluation Criteria in Solid Tumors (RECIST, version 1.1) as complete response (CR), partial response (PR), stable disease (SD), or progressive disease (PD)^1^. Tumor samples were histologically scored by two expert breast pathologists using the Miller-Payne (MP) grading system to confirm pCR.

**RNA sequencing sample preparation**

The TRIzol reagent kit (Invitrogen, Carlsbad, CA, USA) was used for total RNA extraction. RNA quality was evaluated using an Agilent 2100 Bioanalyzer (Agilent Technologies, Palo Alto, CA, USA), followed by RNase-free agarose gel electrophoresis. Oligo (dT) beads were then used to enrich eukaryotic mRNA after total RNA extraction. After fragmenting the enriched mRNAs into short fragments, random primers were used to reverse transcribe them into cDNA. As an alternative to dTTP, dUTP was used to synthesize second-strand cDNA using DNA polymerase I, RNase H, and buffer. Next, cDNA fragments were purified using the QiaQuick PCR extraction kit (Qiagen, Venlo, Netherlands), followed by end repair, poly(A) addition, and ligation to Illumina sequencing adapters. The second-strand cDNA was digested with uracil N-glycosylase (Uracil-N-Glycosylase). The digested products were size-sorted using agarose gel electrophoresis, PCR-amplified, and sequenced using Illumina Nova 6000.

**RNA sequencing data generation**

Raw mRNA sequencing data for each sample were assessed using FastQC v0.12.1 software to ensure the accuracy of subsequent analyses. Raw data were preprocessed using fastp v0.23.4, including the removal of splice sequences and trimming of low-quality reads (main parameters qualified**-**quality**-**phred = 30, length-required = 36). The trimmed clean reads were compared and quantified at the gene level using STAR v2.7.10b software^2^, with the reference genome version Gencode GRCh38,hg38 and two-pass comparison mode two pass Mode = Basic.

**The Cancer Genome Atlas (TCGA) data**

The TCGA breast cancer data were utilized for two parts of our analysis. First, we extrapolated the mRNA-based classification of HR^+^/HER2^+^ breast cancer (BC) from TCGA. Based on the extrapolated results, we validated the differential expression of selected classification markers among the four mRNA-based subtypes using RNA sequencing data from TCGA. Second, for the selection of markers for IHC-based classification, we used RNA sequencing data and protein expression data (protein levels measured with mass spectrometry) from TCGA to examine the correlation between gene mRNA expression and protein expression. Protein expression data measured via mass spectrometry were downloaded from the cBioPortal (http://www.cbioportal.org).

# The Gene Expression Omnibus (GEO) data

In the fourth cohort (GSE196096), 156 patients with HR^+^/HER2^+^ BC were included in microarray datasets. In the fifth cohort (GSE96058), 321 patients with HR^+^/HER2^+^ BC were included in microarray datasets. Gene expression data were normalized based on a prior study. A hybridization probe was mapped to an Entrez Gene ID based on a previous study using the probe with the highest variance when multiple probes were mapped to the same gene ID^3^.

**Based on the expressed unsupervised clustering**

Non-negative matrix factorization (NMF; using the R package “NMF”) was used to identify differentially expressed genes to separate clusters and to determine the optimal clustering number of HR^+^/HER2^+^ breast cancer datasets. Before integrative clustering, all types of data without missing values were required. The expression profile with the top 20% median absolute deviation was retained for further analysis. The parameters are set as follows: nrun=10 and seed=123.

**Selection of markers for IHC-based classification**

First, we performed differential expression analysis on RNA sequencing data and identified highly expressed genes (log2(fold change) ≥ 1, adjusted *P* value < 0.05) in each subtype. Second, using RNA sequencing and mass spectrometry protein expression data from TCGA, we assessed the correlation between the mRNA and protein expression of these genes and retained those that satisfied the following criteria: a correlation coefficient of ≥ 0.7 and a P value of < 0.05. Genes enriched in typical pathways in each subtype are summarized based on the unique biological characteristics of each subtype^4-9^. The expression location and extent of candidate factors corresponding to breast immunohistochemical staining were determined through statistical searches of previous literature and the Human Protein Atlas database. Finally, we integrated the aforementioned data and established a union.

**Immunohistochemistry IHC interpretation and IHC-based classification method**

To estimate the percentage of tumor cells positive for *PFKP*, *GFRA1*, and *KRT5* and their protein expression levels, we immunohistochemically measured the percentage of positive tumor cells (the number of positive tumor cells divided by the total number of tumor cells). All areas containing invasive tumors were assessed during the evaluation of the sections. In contrast, CD8 staining was predominantly found in tumor-infiltrating lymphocytes (TILs). Therefore, we calculated the CD8 protein expression level by counting positively stained cells (the number of positive cells divided by the total number of all types of cells). Immunohistochemistry scores of all stained paraffin-embedded sections were determined independently by two experienced pathologists. This was a blinded study in which pathologists did not have access to the medical records of the patients. Discrepancies between the pathologists were resolved by discussion and consensus. An analysis of the correlation between mRNA-based classification and IHC-based classification was conducted using Cohen's κ statistic^10^. Analyses were performed using version 4.2.2 of the R (https://cran.r-project.org/).

**References**

1.	Eisenhauer, E.A. et al. New response evaluation criteria in solid tumours: revised RECIST guideline (version 1.1). *Eur. J. Cancer* **45**, 228-47 (2009).

2.	Dobin, A. et al. STAR: ultrafast universal RNA-seq aligner. Bioinformatics 29, 15-21 (2013).

3.	Shi, L. et al. The MicroArray Quality Control (MAQC) project shows inter- and intraplatform reproducibility of gene expression measurements. *Nat. Biotechnol.* **24**, 1151-61 (2006).

4.	Chen, J. et al. PFKP alleviates glucose starvation-induced metabolic stress in lung cancer cells via AMPK-ACC2 dependent fatty acid oxidation. *Cell Discov.* **8**, 52 (2022).

5.	Lu, T.J. et al. Phosphofructokinase Platelet Overexpression Accelerated Colorectal Cancer Cell Growth and Motility. *J. Cancer* **14**, 943-951 (2023).

6.	Chen, D.S. & Mellman, I. Elements of cancer immunity and the cancer-immune set point. *Nature* **541**, 321-330 (2017).

7.	Morandi, A. et al. GDNF-RET signaling in ER-positive breast cancers is a key determinant of response and resistance to aromatase inhibitors. *Cancer Res.* **73**, 3783-95 (2013).

8.	Esseghir, S. et al. A role for glial cell derived neurotrophic factor induced expression by inflammatory cytokines and RET/GFR alpha 1 receptor up-regulation in breast cancer. *Cancer Res.* **67**, 11732-41 (2007).

9.	Esseghir, S. et al. Identification of transmembrane proteins as potential prognostic markers and therapeutic targets in breast cancer by a screen for signal sequence encoding transcripts. *J. Pathol.* **210**, 420-30 (2006).

10.	Haibe-Kains, B. et al. A three-gene model to robustly identify breast cancer molecular subtypes. *J. Natl. Cancer Inst.* **104**, 311-25 (2012).

**Supplementary Figures and legends**

**Fig. S1. Extended analysis of molecular biological characterization of the four subtypes.**

**a,** Quality metrics describing the performance of non-negative matrix factorization (NMF) for 2 to 7 clusters.

**b,** The subclass mapping of training cohort and validated subtypes (MUKDEN Validation cohort, GEO GSE96058 cohort, TCGA cohort and GEO I-SPY2 trial cohort). P-value represents the correlation between subtypes, showing that significant associations between subtypes are indicated in red (*p* < 0.05), while differences between subtypes are indicated in blue (*p* > 0.05).

**Fig. S2. Extended analysis of molecular characterization of the four subtypes in the MUKDEN, TCGA and GEO I-SPY2 cohorts.**

**a-c,** Bar graphs depict the Kyoto Encyclopedia of Genes and Genomes (KEGG) pathway enrichment related to the biological functions of signature genes for each mRNA subtype in the MUKDEN, TCGA and GEO I-SPY2 cohorts.

**Fig. S3. Extended analysis of molecular characterization of the four subtypes in the MUKDEN validation and GSE96058 cohorts.**

**a-b,** Bar graphs depict the KEGG pathway enrichment related to the biological functions of signature genes for each mRNA subtype in the MUKDEN validation and GSE96058 cohorts.

**Fig. S4. Extended analysis of clinical status of the four subtypes.**

**a,** Association of MUKDEN subtypes with different clinical statuses. P-values were obtained using the two-sided Fisher’s exact test.

**b,** Bar graph of pathologic complete response (pCR) rates for each subtype in MUKDEN validation cohort.

**Fig. S5. Extended analysis of efficacy characteristics of treatment regimes.**

**a–c,** Volcano plot of differential genes between pCR and non-pCR patients with three treatment regimens. a, Chemo + Tar (chemotherapy plus anti-HER2-targeted therapy); b, ET + CDKi + Tar (endocrine therapy, CDK4/6 inhibitor, and anti-HER2-targeted therapy); c, ADC + TKI (anti**-**HER2 antibody**-**drug conjugate combined with tyrosine kinase inhibitor).

**d,** The gene set enrichment analysis (GSEA) of ER, Drug metabolism-Cytochrome P450, and Primary immunodeficiency signaling pathway in ET + CDKi +Tar subgroup.

**e,** The GSEA enrichment analysis of HIF-1, NK cell mediated cytotoxicity, and Primary immunodeficiency singaling pathway in ADC+TKI subgroup.

**Fig. S6. Extended analysis of MUKDEN I feature factors in external validation data.**

**a and b,** Boxplot showing the expression levels (normalized) of HIF-1 signaling pathway genes (*ERBB2*, *CASP14*, *PFKP*, and *HIF1A*) among four subtypes in validation cohorts. Boxplots show median (central line), upper and lower quartiles (box limits), 1.5× interquartile range (whiskers).

**Fig. S7. Extended analysis of Estrogen Receptor (ER) signaling pathway**

**a,** The GSEA enrichment analysis of ER, Cell Cycle and PD-L1 check point signaling pathway in MUKDEN II subtype cohort.

**b and c,** Boxplot showing the expression levels (normalized) of ER and Cell Cycle signaling pathway genes among four subtypes in validation cohorts. Boxplots show median (central line), upper and lower quartiles (box limits), 1.5× interquartile range (whiskers).

**Fig. S8. Extended analysis of immune infiltration in external validation data.**

**a-d**, Heatmap showing the estimated abundance of 10 microenvironmental cell types (MCP Counter) among the four MUKDEN subtypes in the TCGA (a), GEO I-SPY2 (b), MUKDEN Validation (c) and GEO GSE96058 (d) cohorts. The color represents the expression of the microenvironment cell, high expression is shown in red, while low expression is shown in blue.

**e,** Boxplot showing the expression levels of five immune checkpoint genes among four subtypes. Boxplots show median (central line), upper and lower quartiles (box limits), 1.5× interquartile range (whiskers).

**Fig. S9. Extended analysis of immune and stromal cell populations in the four subtypes.**

**a,** Box plot of the abundance of immune and stromal cell populations distinguished by different subtypes.

**b,** Boxplot of immune and stromal scores from ESTIMATE for the four subtypes. Boxplots show median (central line), upper and lower quartiles (box limits), 1.5× interquartile range (whiskers).

**Fig. S10. Extended analysis of highly heterogeneous characteristics.**

**a and b,** Boxplot showing the expression levels (normalized) of the PI3K-AKT, ECM, Wnt, and EGFR signaling pathways genes among four subtypes in validation cohorts. Boxplots show median (central line), upper and lower quartiles (box limits), 1.5× interquartile range (whiskers).

**Fig. S11. Correlation between the PI3K-AKT and ECM signaling pathways in each MUKDEN subtype.**

**a-d,** Correlation between the PI3K-AKT and ECM signaling pathways in each MUKDEN subtype. The enrichment score for each sample in each gene set was calculated using the single-sample GSEA (ssGSEA) algorithm.

**Fig. S12. Extended analysis of workflow for marker selection for immunohistochemistry-based HR^+^/HER2^+^ BC classification.**

**a,** Workflow for marker selection for immunohistochemistry-based HR^+^/HER2^+^BC classification.

**b,** Boxplot showing the mRNA expression of *PFKP*, *GFRA1*, *CD8A*, and *KRT5* among four subtypes. Boxplots show median (central line), upper and lower quartiles (box limits), 1.5× interquartile range (whiskers).

**c,** Correlation between protein expression and mRNA expression of the four factors.

**Fig. S13. Summary figure of MUKDEN subtypes and precision treatment for HR^+^/HER2^+^ breast cancer.**

**a,** A summary figure showing the overview of MUKDEN subtypes, including molecular characteristics and treatment strategies.

**Table S1. MUKDEN Training Cohort information**

| **Patient Code** | **MUKDENsubtype** | | **PAM50** | | **RNAseq** | **Sex** | **Age** | **Menopause** | **Grade** | **pT** | **pN** | **Clinical Stage** | **ER_**  **percent** | **ER_**  **status** | **PR_**  **percent** | **PR_**  **status** | **HER2 IHC Status** | **HER2_FISH Status** | **Ki67** | **Neoadjuvant Therapy** | **Therapy Arm** | **pCR** |
| --- | --- | --- | --- | --- | --- | --- | --- | --- | --- | --- | --- | --- | --- | --- | --- | --- | --- | --- | --- | --- | --- | --- |
| SUXJ | Ⅰ | Her2 | | | Yes | Female | 54 | YES | 2 | 2 | 1 | IIB | 60 | Positive | 0 | Negative | 3+ | Unknown | 35 | YES | ET+CDKi+Tar | pCR |
| SJIX | Ⅰ | Her2 | | Yes | | Female | 54 | YES | 2 | 2 | 1 | IIB | 60 | Positive | 0 | Negative | 3+ | Unknown | 35 | YES | ET+CDKi+Tar | pCR |
| WAGF | Ⅳ | Normal | | Yes | | Female | 53 | YES | 2 | 2 | 0 | IIA | 0 | Negative | 10 | Positive | 2+ | Positive | 20 | YES | Chemo+Tar | pCR |
| CHJM | Ⅲ | Basal | | Yes | | Female | 66 | YES | 2 | 2 | 1 | IIB | 90 | Positive | 0 | Negative | 2+ | Positive | 30 | YES | Chemo+Tar | non-pCR |
| CHLY | Ⅱ | LumA | | Yes | | Female | 57 | YES | 2 | 2 | 0 | IIA | 100 | Positive | 80 | Positive | 2+ | Positive | 10 | YES | ADC+TKI | non-pCR |
| CLIY | Ⅱ | LumA | | Yes | | Female | 57 | YES | 2 | 2 | 0 | IIA | 100 | Positive | 80 | Positive | 2+ | Positive | 10 | YES | ADC+TKI | non-pCR |
| CUYH | Ⅰ | Her2 | | Yes | | Female | 57 | YES | 2 | 3 | 2 | IIIA | 30 | Positive | 5 | Positive | 3+ | Unknown | 70 | YES | ADC+TKI | non-pCR |
| FUCF | Ⅲ | Her2 | | Yes | | Female | 55 | YES | 2 | 2 | 2 | IIIA | 60 | Positive | 0 | Negative | 3+ | Unknown | 30 | YES | Chemo+Tar | non-pCR |
| GAOH | Ⅲ | Her2 | | Yes | | Female | 52 | YES | 2 | 2 | 1 | IIB | 0 | Negative | 10 | Positive | 3+ | Unknown | 70 | YES | Chemo+Tar | pCR |
| LIJY | Ⅰ | Her2 | | Yes | | Female | 54 | YES | 2 | 2 | 0 | IIA | 40 | Positive | 0 | Negative | 3+ | Unknown | 60 | YES | ADC+TKI | pCR |
| LINN | Ⅰ | Her2 | | Yes | | Female | 39 | NO | 2 | 2 | 1 | IIB | 60 | Positive | 20 | Positive | 3+ | Unknown | 50 | YES | ADC+TKI | non-pCR |
| LIXL | Ⅱ | LumB | | Yes | | Female | 43 | NO | 2 | 2 | 1 | IIB | 95 | Positive | 40 | Positive | 3+ | Unknown | 30 | YES | ADC+TKI | non-pCR |
| LIYH | Ⅱ | Her2 | | Yes | | Female | 50 | NO | 2 | 3 | 2 | IIIA | 70 | Positive | 10 | Positive | 3+ | Unknown | 30 | YES | ADC+TKI | pCR |
| LIYL | Ⅰ | Her2 | | Yes | | Female | 58 | YES | 2 | 2 | 2 | IIIA | 50 | Positive | 5 | Positive | 3+ | Unknown | 70 | YES | ADC+TKI | pCR |
| LIHO | Ⅲ | Basal | | Yes | | Female | 37 | NO | 2 | 2 | 2 | IIIA | 70 | Positive | 25 | Positive | 3+ | Unknown | 70 | YES | ADC+TKI | pCR |
| LISQ | Ⅲ | Her2 | | Yes | | Female | 54 | YES | 2 | 2 | 0 | IIA | 0 | Negative | 10 | Positive | 3+ | Unknown | 60 | YES | Chemo+Tar | non-pCR |
| LIYP | Ⅱ | LumB | | Yes | | Female | 59 | YES | 2 | 2 | 2 | IIIA | 90 | Positive | 20 | Positive | 3+ | Unknown | 30 | YES | ADC+TKI | non-pCR |
| LUJN | Ⅲ | Her2 | | Yes | | Female | 40 | NO | 2 | 2 | 1 | IIB | 70 | Positive | 30 | Positive | 2+ | Positive | 65 | YES | ADC+TKI | pCR |
| MAXX | Ⅲ | Her2 | | Yes | | Female | 60 | YES | 2 | 2 | 1 | IIB | 5 | Positive | 15 | Positive | 3+ | Unknown | 75 | YES | Chemo+Tar | pCR |
| MEXW | Ⅱ | LumB | | Yes | | Female | 49 | YES | 2 | 2 | 2 | IIIA | 90 | Positive | 90 | Positive | 2+ | Positive | 30 | YES | ADC+TKI | non-pCR |

| **Patient Code** | **MUKDE_**  **subtype** | **PAM50** | **RNA_**  **Seq** | **Sex** | **Age** | **Menopause** | **Grade** | **pT** | **pN** | **Clinical Stage** | **ER_ percent** | **ER_**  **status** | **PR_**  **percent** | **PR_**  **status** | **HER2_IHC Status** | **HER2_FISH Status** | **Ki67** | **Neoadjuvant Therapy** | **Therapy Arm** | **pCR** |
| --- | --- | --- | --- | --- | --- | --- | --- | --- | --- | --- | --- | --- | --- | --- | --- | --- | --- | --- | --- | --- |
| RESM | Ⅳ | LumB | Yes | Female | 52 | YES | 2 | 1 | 2 | IIIA | 90 | Positive | 70 | Positive | 2+ | Positive | 20 | YES | Chemo+Tar | pCR |
| SUMC | Ⅳ | LumA | Yes | Female | 32 | NO | 2 | 2 | 2 | IIIA | 95 | Positive | 95 | Positive | 3+ | Unknown | 40 | YES | Chemo+Tar | pCR |
| SULI | Ⅰ | Her2 | Yes | Female | 34 | NO | 2 | 2 | 2 | IIIA | 70 | Positive | 5 | Positive | 2+ | Positive | 40 | YES | ADC+TKI | pCR |
| SUXI | Ⅰ | Her2 | Yes | Female | 57 | YES | 2 | 2 | 0 | IIA | 90 | Positive | 0 | Negative | 2+ | Positive | 30 | YES | Chemo+Tar | pCR |
| SUYJA | Ⅰ | Her2 | Yes | Female | 50 | NO | 2 | 2 | 0 | IIA | 70 | Positive | 40 | Positive | 3+ | Unknown | 50 | YES | ADC+TKI | pCR |
| WAXH | Ⅳ | Normal | Yes | Female | 52 | YES | 2 | 2 | 0 | IIA | 10 | Positive | 10 | Positive | 2+ | Positive | 20 | YES | Chemo+Tar | pCR |
| TAGM | Ⅲ | Her2 | Yes | Female | 52 | YES | 2 | 2 | 2 | IIIA | 30 | Positive | 0 | Negative | 3+ | Unknown | 40 | YES | ADC+TKI | pCR |
| TIXI | Ⅱ | LumB | Yes | Female | 27 | NO | 2 | 2 | 0 | IIA | 80 | Positive | 40 | Positive | 2+ | Positive | 40 | YES | ADC+TKI | non-pCR |
| TAXN | Ⅱ | LumB | Yes | Female | 27 | NO | 2 | 2 | 0 | IIA | 80 | Positive | 40 | Positive | 2+ | Positive | 40 | YES | ADC+TKI | non-pCR |
| WAYI | Ⅲ | Her2 | Yes | Female | 55 | YES | 2 | 3 | 2 | IIIA | 10 | Positive | 0 | Negative | 3+ | Unknown | 40 | YES | ADC+TKI | pCR |
| XUYF | Ⅰ | Her2 | Yes | Female | 68 | YES | 2 | 2 | 0 | IIA | 0 | Negative | 10 | Positive | 3+ | Unknown | 30 | YES | Chemo+Tar | non-pCR |
| YAJY | Ⅱ | Her2 | Yes | Female | 48 | NO | 2 | 2 | 0 | IIA | 90 | Positive | 90 | Positive | 2+ | Positive | 40 | YES | ADC+TKI | pCR |
| YAXC | Ⅰ | Her2 | Yes | Female | 43 | NO | 2 | 2 | 1 | IIIA | 80 | Positive | 20 | Positive | 2+ | Positive | 20 | YES | Chemo+Tar | pCR |
| YAXF | Ⅲ | Her2 | Yes | Female | 54 | YES | 2 | 2 | 2 | IIIA | 10 | Positive | 0 | Negative | 2+ | Positive | 20 | YES | ADC+TKI | pCR |
| ZAGH | Ⅱ | Basal | Yes | Female | 37 | NO | 2 | 2 | 3 | IIIC | 80 | Positive | 60 | Positive | 3+ | Unknown | 50 | YES | ADC+TKI | pCR |
| ZHYB | Ⅰ | Her2 | Yes | Female | 53 | YES | 2 | 2 | 2 | IIIA | 0 | Negative | 10 | Positive | 3+ | Unknown | 70 | YES | ADC+TKI | pCR |
| ZHFY | Ⅰ | Her2 | Yes | Female | 46 | NO | 2 | 2 | 2 | IIIA | 30 | Positive | 50 | Positive | 2+ | Positive | 5 | YES | Chemo+Tar | pCR |
| BAYY | Ⅱ | LumB | Yes | Female | 69 | YES | 3 | 1 | 1 | IIA | 95 | Positive | 3 | Positive | 3+ | Unknown | 30 | NO | Adjuvant Therapy | Adjuvant Therapy |
| CHJU | Ⅱ | LumB | Yes | Female | 59 | YES | 2 | 2 | 1 | IIB | 80 | Positive | 40 | Positive | 2+ | Positive | 50 | NO | Adjuvant Therapy | Adjuvant Therapy |
| DUMQ | Ⅳ | Normal | Yes | Female | 41 | NO | 2 | 1 | 0 | IA | 30 | Positive | 30 | Positive | 3+ | Unknown | 60 | NO | Adjuvant Therapy | Adjuvant Therapy |
| GUYZ | Ⅲ | LumB | Yes | Female | 65 | YES | 2 | 1 | 0 | IA | 100 | Positive | 0 | Negative | 3+ | Unknown | 30 | NO | Adjuvant Therapy | Adjuvant Therapy |
| LAXP | Ⅱ | LumB | Yes | Female | 47 | YES | 2 | 2 | 1 | IIB | 80 | Positive | 30 | Positive | 2+ | Positive | 70 | YES | Chemo+Tar | pCR |
| **Patient Code** | **MUKDE_**  **subtype** | **PAM50** | **RNA_**  **Seq** | **Sex** | **Age** | **Menopause** | **Grade** | **pT** | **pN** | **Clinical Stage** | **ER_ percent** | **ER_**  **status** | **PR_**  **percent** | **PR_**  **status** | **HER2_IHC Status** | **HER2_FISH Status** | **Ki67** | **Neoadjuvant Therapy** | **Therapy Arm** | **pCR** |
| LISY | Ⅱ | LumB | Yes | Female | 57 | YES | 2 | 1 | 0 | IA | 100 | Positive | 0 | Negative | 3+ | Unknown | 30 | NO | Adjuvant Therapy | Adjuvant Therapy |
| LISH | Ⅳ | Her2 | Yes | Female | 35 | NO | 2 | 1 | 0 | IA | 40 | Positive | 40 | Positive | 3+ | Unknown | 40 | NO | Adjuvant Therapy | Adjuvant Therapy |
| LILI | Ⅳ | Basal | Yes | Female | 44 | NO | 2 | 2 | 0 | IIA | 90 | Positive | 50 | Positive | 3+ | Unknown | 70 | YES | ET+CDKi+Tar | pCR |
| MEFH | Ⅰ | Her2 | Yes | Female | 60 | YES | 2 | 2 | 0 | IIA | 60 | Positive | 5 | Positive | 2+ | Positive | 60 | YES | ET+CDKi+Tar | pCR |
| POYL | Ⅳ | LumA | Yes | Female | 39 | NO | 2 | 2 | 1 | IIB | 30 | Positive | 40 | Positive | 2+ | Positive | 5 | YES | ADC+TKI | non-pCR |
| QULJ | Ⅲ | LumB | Yes | Female | 58 | YES | 2 | 2 | 0 | IIA | 90 | Positive | 60 | Positive | 2+ | Positive | 15 | YES | ET+CDKi+Tar | pCR |
| SHRM | Ⅳ | Basal | Yes | Female | 50 | NO | 2 | 2 | 0 | IIA | 60 | Positive | 90 | Positive | 3+ | Unknown | 70 | YES | ET+CDKi+Tar | non-pCR |
| TOLI | Ⅱ | Basal | Yes | Female | 61 | YES | 2 | 2 | 1 | IIB | 80 | Positive | 30 | Positive | 2+ | Positive | 30 | YES | ET+CDKi+Tar | pCR |
| WAFY | Ⅳ | Normal | Yes | Female | 69 | YES | 2 | 2 | 0 | IIA | 80 | Positive | 80 | Positive | 2+ | Positive | 35 | YES | Chemo+Tar | non-pCR |
| WAHY | Ⅰ | Her2 | Yes | Female | 60 | YES | 2 | 2 | 0 | IIA | 100 | Positive | 40 | Positive | 3+ | Unknown | 30 | YES | ET+CDKi+Tar | non-pCR |
| WAXY | Ⅰ | Her2 | Yes | Female | 51 | YES | 2 | 3 | 1 | IIIA | 60 | Positive | 0 | Negative | 3+ | Unknown | 30 | YES | ET+CDKi+Tar | non-pCR |
| WUTA | Ⅰ | Her2 | Yes | Female | 28 | NO | 2 | 2 | 1 | IIB | 70 | Positive | 5 | Positive | 3+ | Unknown | 60 | YES | ET+CDKi+Tar | pCR |
| YAXD | Ⅱ | LumB | Yes | Female | 43 | NO | 2 | 2 | 0 | IIA | 100 | Positive | 90 | Positive | 2+ | Positive | 40 | YES | Chemo+Tar | non-pCR |
| YAJA | Ⅱ | Her2 | Yes | Female | 53 | YES | 3 | 2 | 0 | IIA | 30 | Positive | 70 | Positive | 3+ | Unknown | 50 | NO | Adjuvant Therapy | Adjuvant Therapy |
| YUJM | Ⅲ | Her2 | Yes | Female | 57 | YES | 2 | 1 | 0 | IA | 80 | Positive | 80 | Positive | 3+ | Unknown | 60 | NO | Adjuvant Therapy | Adjuvant Therapy |
| ZHKF | Ⅲ | LumA | Yes | Female | 43 | NO | 2 | 1 | 0 | IA | 0.8 | Positive | 40 | Positive | 3+ | Unknown | 40 | NO | Adjuvant Therapy | Adjuvant Therapy |
| ZHXL | Ⅲ | Her2 | Yes | Female | 55 | YES | 2 | 2 | 0 | IIA | 80 | Positive | 40 | Positive | 3+ | Unknown | 40 | NO | Adjuvant Therapy | Adjuvant Therapy |
| ZHLM | Ⅱ | Her2 | Yes | Female | 67 | YES | 2 | 1 | 1 | IIA | 90 | Positive | 30 | Positive | 3+ | Unknown | 30 | YES | ET+CDKi+Tar | non-pCR |
| CHAL | Ⅳ | Her2 | Yes | Female | 35 | NO | 2 | 2 | 1 | IIB | 90 | Positive | 80 | Positive | 3+ | Unknown | 75 | NO | Adjuvant Therapy | Adjuvant Therapy |
| CHGU | Ⅱ | LumB | Yes | Female | 57 | YES | 2 | 1 | 3 | IIIC | 95 | Positive | 5 | Positive | 2+ | Positive | 30 | NO | Adjuvant Therapy | Adjuvant Therapy |
| DELN | Ⅰ | Her2 | Yes | Female | 42 | NO | 2 | 2 | 0 | IIA | 50 | Positive | 10 | Positive | 3+ | Unknown | 30 | NO | Adjuvant Therapy | Adjuvant Therapy |
| DULJ | Ⅳ | LumA | Yes | Female | 59 | YES | 2 | 2 | 1 | IIB | 95 | Positive | 95 | Positive | 2+ | Positive | 20 | NO | Adjuvant Therapy | Adjuvant Therapy |
| GUGH | Ⅲ | Basal | Yes | Female | 62 | YES | 3 | 2 | 0 | IIA | 95 | Positive | 50 | Positive | 2+ | Positive | 60 | NO | Adjuvant Therapy | Adjuvant Therapy |
| **Patient Code** | **MUKDE_**  **subtype** | **PAM50** | **RNA_**  **Seq** | **Sex** | **Age** | **Menopause** | **Grade** | **pT** | **pN** | **Clinical Stage** | **ER_ percent** | **ER_**  **status** | **PR_**  **percent** | **PR_**  **status** | **HER2_IHC Status** | **HER2_FISH Status** | **Ki67** | **Neoadjuvant Therapy** | **Therapy Arm** | **pCR** |
| HULJ | Ⅰ | Her2 | Yes | Female | 48 | NO | 2 | 2 | 2 | IIIA | 80 | Positive | 90 | Positive | 2+ | Positive | 20 | NO | Adjuvant Therapy | Adjuvant Therapy |
| HUYX | Ⅱ | LumB | Yes | Female | 56 | YES | 2 | 2 | 2 | IIIA | 90 | Positive | 2 | Positive | 2+ | Positive | 60 | NO | Adjuvant Therapy | Adjuvant Therapy |
| HUYF | Ⅱ | LumB | Yes | Female | 49 | YES | 2 | 2 | 0 | IIA | 95 | Positive | 70 | Positive | 2+ | Positive | 30 | NO | Adjuvant Therapy | Adjuvant Therapy |
| LISLA | Ⅳ | LumA | Yes | Female | 81 | YES | 2 | 2 | 0 | IIA | 95 | Positive | 95 | Positive | 2+ | Positive | 5 | NO | Adjuvant Therapy | Adjuvant Therapy |
| LISR | Ⅰ | Her2 | Yes | Female | 43 | NO | 2 | 2 | 2 | IIIA | 20 | Positive | 5 | Positive | 3+ | Unknown | 20 | NO | Adjuvant Therapy | Adjuvant Therapy |
| LIYX | Ⅰ | Her2 | Yes | Female | 52 | YES | 2 | 2 | 1 | IIB | 80 | Positive | 30 | Positive | 2+ | Positive | 30 | NO | Adjuvant Therapy | Adjuvant Therapy |
| MAML | Ⅲ | LumB | Yes | Female | 32 | NO | 2 | 2 | 0 | IIA | 95 | Positive | 70 | Positive | 2+ | Positive | 60 | NO | Adjuvant Therapy | Adjuvant Therapy |
| MAXPA | Ⅱ | LumB | Yes | Female | 63 | YES | 2 | 1 | 0 | IA | 95 | Positive | 75 | Positive | 2+ | Positive | 30 | NO | Adjuvant Therapy | Adjuvant Therapy |
| REXH | Ⅳ | LumA | Yes | Female | 38 | NO | 2 | 1 | 0 | IA | 90 | Positive | 90 | Positive | 2+ | Positive | 20 | NO | Adjuvant Therapy | Adjuvant Therapy |
| SUYJ | Ⅱ | Her2 | Yes | Female | 68 | YES | 2 | 1 | 0 | IA | 95 | Positive | 80 | Positive | 3+ | Unknown | 40 | NO | Adjuvant Therapy | Adjuvant Therapy |
| WAZH | Ⅳ | LumA | Yes | Female | 43 | NO | 2 | 2 | 0 | IIA | 90 | Positive | 90 | Positive | 2+ | Positive | 5 | NO | Adjuvant Therapy | Adjuvant Therapy |
| WAYH | Ⅲ | LumB | Yes | Female | 57 | YES | 3 | 2 | 0 | IIA | 95 | Positive | 20 | Positive | 2+ | Positive | 60 | NO | Adjuvant Therapy | Adjuvant Therapy |
| XUGF | Ⅱ | LumB | Yes | Female | 73 | YES | 2 | 2 | 1 | IIB | 90 | Positive | 50 | Positive | 2+ | Positive | 65 | NO | Adjuvant Therapy | Adjuvant Therapy |
| YAZY | Ⅱ | LumA | Yes | Female | 67 | YES | 2 | 2 | 0 | IIA | 90 | Positive | 90 | Positive | 2+ | Positive | 30 | NO | Adjuvant Therapy | Adjuvant Therapy |
| ZHGQ | Ⅱ | LumB | Yes | Female | 64 | YES | 2 | 1 | 0 | IA | 80 | Positive | 10 | Positive | 2+ | Positive | 40 | NO | Adjuvant Therapy | Adjuvant Therapy |
| ZHXJ | Ⅱ | LumB | Yes | Female | 42 | NO | 2 | 2 | 1 | IIB | 90 | Positive | 70 | Positive | 2+ | Positive | 40 | NO | Adjuvant Therapy | Adjuvant Therapy |
| ZHAY | Ⅲ | Basal | Yes | Female | 39 | NO | 2 | 1 | 1 | IIA | 95 | Positive | 95 | Positive | 2+ | Positive | 40 | NO | Adjuvant Therapy | Adjuvant Therapy |
| ZHXX | Ⅳ | Normal | Yes | Female | 58 | YES | 2 | 2 | 2 | IIIA | 95 | Positive | 20 | Positive | 3+ | Unknown | 25 | NO | Adjuvant Therapy | Adjuvant Therapy |
| GEYH | Ⅱ | Normal | Yes | Female | 43 | NO | 2 | 1 | 1 | IIA | 90 | Positive | 90 | Positive | 2+ | Positive | 30 | NO | Adjuvant Therapy | Adjuvant Therapy |
| SHHB | Ⅰ | Her2 | Yes | Female | 34 | NO | 2 | 2 | 0 | IIA | 80 | Positive | 40 | Positive | 3+ | Unknown | 90 | YES | ADC+TKI | non-pCR |
| HEJI | Ⅳ | Normal | Yes | Female | 45 | NO | 2 | 2 | 0 | IIA | 50 | Positive | 80 | Positive | 3+ | Unknown | 20 | YES | Chemo+Tar | non-pCR |
| QULP | Ⅱ | LumB | Yes | Female | 61 | YES | 2 | 2 | 2 | IIIA | 40 | Positive | 40 | Positive | 2+ | Positive | 30 | YES | Chemo+Tar | pCR |
| **Patient Code** | **MUKDE_**  **subtype** | **PAM50** | **RNA_**  **Seq** | **Sex** | **Age** | **Menopause** | **Grade** | **pT** | **pN** | **Clinical Stage** | **ER_ percent** | **ER_**  **status** | **PR_**  **percent** | **PR_**  **status** | **HER2_IHC Status** | **HER2_FISH Status** | **Ki67** | **Neoadjuvant Therapy** | **Therapy Arm** | **pCR** |
| SOWY | Ⅱ | LumB | Yes | Female | 58 | YES | 2 | 2 | 1 | IIB | 60 | Positive | 40 | Positive | 2+ | Positive | 30 | YES | ADC+TKI | pCR |
| ZHWE | Ⅳ | Normal | Yes | Female | 50 | NO | 2 | 2 | 0 | IIA | 90 | Positive | 50 | Positive | 3+ | Unknown | 30 | YES | ADC+TKI | pCR |
| MAHM | Ⅲ | Her2 | Yes | Female | 53 | YES | 2 | 2 | 0 | IIA | 10 | Positive | 0 | Negative | 3+ | Unknown | 30 | YES | Chemo+Tar | pCR |
| LISL | Ⅰ | Her2 | Yes | Female | 47 | YES | 2 | 2 | 2 | IIIA | 80 | Positive | 2 | Positive | 3+ | Unknown | 20 | NO | Adjuvant Therapy | Adjuvant Therapy |
| LAFR | Ⅲ | LumA | Yes | Female | 71 | YES | 2 | 2 | 3 | IIIC | 95 | Positive | 95 | Positive | 2+ | Positive | 40 | YES | Chemo+Tar | non-pCR |
| LIYC | Ⅲ | Her2 | Yes | Female | 69 | YES | 2 | 2 | 1 | IIB | 70 | Positive | 10 | Positive | 2+ | Positive | 30 | YES | ET+CDKi+Tar | pCR |
| CHHO | Ⅱ | LumB | Yes | Female | 38 | NO | 2 | 2 | 1 | IIB | 90 | Positive | 50 | Positive | 2+ | Positive | 30 | YES | ADC+TKI | non-pCR |
| DABR | Ⅲ | Her2 | Yes | Female | 71 | YES | 2 | 1 | 1 | IIA | 60 | Positive | 60 | Positive | 2+ | Positive | 60 | YES | ET+CDKi+Tar | non-pCR |
| FULX | Ⅰ | Basal | Yes | Female | 59 | YES | 2 | 2 | 0 | IIA | 99 | Positive | 90 | Positive | 3+ | Unknown | 50 | YES | ET+CDKi+Tar | non-pCR |
| GUJY | Ⅲ | LumB | Yes | Female | 61 | YES | 2 | 2 | 0 | IIA | 95 | Positive | 20 | Positive | 2+ | Positive | 80 | YES | ET+CDKi+Tar | non-pCR |
| HERM | Ⅳ | LumA | Yes | Female | 30 | NO | 2 | 2 | 0 | IIA | 50 | Positive | 10 | Positive | 3+ | Unknown | 50 | YES | ET+CDKi+Tar | pCR |
| JILL | Ⅰ | LumB | Yes | Female | 39 | NO | 2 | 3 | 1 | IIIA | 90 | Positive | 95 | Positive | 3+ | Unknown | 30 | YES | ET+CDKi+Tar | pCR |
| JIRU | Ⅲ | Basal | Yes | Female | 41 | NO | 2 | 2 | 0 | IIA | 40 | Positive | 40 | Positive | 3+ | Unknown | 40 | YES | ET+CDKi+Tar | non-pCR |
| LIJF | Ⅲ | LumA | Yes | Female | 58 | YES | 2 | 2 | 0 | IIA | 90 | Positive | 0 | Negative | 2+ | Positive | 30 | YES | ET+CDKi+Tar | non-pCR |
| SOHF | Ⅰ | LumB | Yes | Female | 51 | YES | 2 | 2 | 0 | IIA | 90 | Positive | 70 | Positive | 2+ | Positive | 30 | YES | ET+CDKi+Tar | non-pCR |
| WULM | Ⅱ | LumB | Yes | Female | 61 | YES | 2 | 2 | 0 | IIA | 99 | Positive | 5 | Positive | 2+ | Positive | 20 | YES | ET+CDKi+Tar | non-pCR |
| XUGY | Ⅰ | LumB | Yes | Female | 65 | YES | 2 | 2 | 1 | IIB | 70 | Positive | 70 | Positive | 2+ | Positive | 20 | YES | ET+CDKi+Tar | non-pCR |
| XUNA | Ⅱ | Basal | Yes | Female | 31 | NO | 2 | 2 | 1 | IIB | 55 | Positive | 5 | Positive | 3+ | Unknown | 40 | YES | ET+CDKi+Tar | pCR |
| SXHA | Ⅰ | Her2 | Yes | Female | 41 | NO | 2 | 1 | 1 | IIA | 90 | Positive | 1 | Positive | 2+ | Positive | 30 | YES | Chemo+Tar | non-pCR |
| PFZH | Ⅰ | Her2 | Yes | Female | 55 | YES | 2 | 3 | 1 | IIIA | 30 | Positive | 5 | Positive | 2+ | Positive | 25 | YES | Chemo+Tar | non-pCR |
| WAWW | Ⅰ | LumB | Yes | Female | 50 | NO | 2 | 3 | 1 | IIIA | 70 | Positive | 50 | Positive | 2+ | Positive | 60 | YES | Chemo+Tar | non-pCR |
| ZCNX | Ⅳ | Basal | Yes | Female | 55 | YES | 2 | 2 | 0 | IIA | 90 | Positive | 5 | Positive | 2+ | Positive | 40 | YES | Chemo+Tar | pCR |
| **Patient Code** | **MUKDE_**  **subtype** | **PAM50** | **RNA_**  **Seq** | **Sex** | **Age** | **Menopause** | **Grade** | **pT** | **pN** | **Clinical Stage** | **ER_ percent** | **ER_**  **status** | **PR_**  **percent** | **PR_**  **status** | **HER2_IHC Status** | **HER2_FISH Status** | **Ki67** | **Neoadjuvant Therapy** | **Therapy Arm** | **pCR** |
| MAQI | Ⅱ | LumA | Yes | Female | 49 | YES | 2 | 2 | 0 | IIA | 60 | Positive | 5 | Positive | 2+ | Positive | 70 | YES | Chemo+Tar | non-pCR |
| MRFF | Ⅰ | Her2 | Yes | Female | 51 | YES | 2 | 1 | 1 | IIA | 70 | Positive | 15 | Positive | 3+ | Unknown | 40 | YES | Chemo+Tar | non-pCR |
| WULI | Ⅱ | LumB | Yes | Female | 29 | NO | 2 | 2 | 0 | IIA | 30 | Positive | 90 | Positive | 3+ | Unknown | 20 | YES | Chemo+Tar | pCR |
| HAYY | Ⅲ | Basal | Yes | Female | 45 | NO | 2 | 1 | 1 | IIA | 30 | Positive | 20 | Positive | 3+ | Unknown | 20 | YES | Chemo+Tar | non-pCR |
| CUXP | Ⅰ | Her2 | Yes | Female | 49 | YES | 2 | 2 | 0 | IIA | 10 | Positive | 5 | Positive | 3+ | Unknown | 40 | YES | Chemo+Tar | pCR |
| ZHSY | Ⅲ | Her2 | Yes | Female | 60 | YES | 2 | 2 | 0 | IIA | 80 | Positive | 20 | Positive | 3+ | Unknown | 50 | YES | Chemo+Tar | non-pCR |
| JIGL | Ⅰ | LumB | Yes | Female | 60 | YES | 2 | 1 | 1 | IIA | 70 | Positive | 40 | Positive | 2+ | Positive | 60 | YES | Chemo+Tar | non-pCR |
| HAXF | Ⅰ | Basal | Yes | Female | 69 | YES | 2 | 2 | 1 | IIIA | 0 | Negative | 10 | Positive | 2+ | Positive | 35 | YES | Chemo+Tar | non-pCR |
| JIXL | Ⅲ | Her2 | Yes | Female | 29 | NO | 2 | 1 | 1 | IIA | 40 | Positive | 10 | Positive | 3+ | Unknown | 40 | YES | Chemo+Tar | non-pCR |
| NAHN | Ⅲ | LumB | Yes | Female | 25 | NO | 2 | 2 | 0 | IIA | 70 | Positive | 10 | Positive | 3+ | Unknown | 25 | YES | Chemo+Tar | non-pCR |
| LIYY | Ⅰ | Her2 | Yes | Female | 47 | YES | 2 | 2 | 0 | IIA | 70 | Positive | 5 | Positive | 3+ | Unknown | 30 | YES | Chemo+Tar | non-pCR |
| ZHCX | Ⅳ | LumA | Yes | Female | 50 | NO | 2 | 2 | 0 | IIA | 90 | Positive | 80 | Positive | 3+ | Unknown | 50 | YES | Chemo+Tar | non-pCR |
| WAGL | Ⅰ | LumB | Yes | Female | 50 | NO | 2 | 1 | 1 | IIA | 80 | Positive | 50 | Positive | 2+ | Positive | 30 | YES | Chemo+Tar | non-pCR |
| BUXP | Ⅰ | Her2 | Yes | Female | 41 | NO | 2 | 2 | 0 | IIA | 50 | Positive | 30 | Positive | 3+ | Unknown | 30 | YES | Chemo+Tar | non-pCR |
| FUXY | Ⅳ | Basal | Yes | Female | 59 | YES | 2 | 1 | 1 | IIA | 20 | Positive | 10 | Positive | 3+ | Unknown | 30 | YES | Chemo+Tar | non-pCR |
| WUXL | Ⅰ | Basal | Yes | Female | 54 | YES | 2 | 1 | 1 | IIA | 80 | Positive | 70 | Positive | 2+ | Positive | 20 | YES | Chemo+Tar | non-pCR |
| WACH | Ⅰ | Her2 | Yes | Female | 50 | NO | 2 | 2 | 0 | IIA | 10 | Positive | 60 | Positive | 3+ | Unknown | 40 | YES | Chemo+Tar | non-pCR |
| YULH | Ⅱ | Basal | Yes | Female | 59 | YES | 2 | 1 | 1 | IIA | 90 | Positive | 70 | Positive | 2+ | Positive | 60 | YES | Chemo+Tar | non-pCR |
| XUSY | Ⅱ | LumB | Yes | Female | 40 | NO | 2 | 2 | 0 | IIA | 45 | Positive | 10 | Negative | 3+ | Unknown | 10 | YES | Chemo+Tar | non-pCR |
| MAXP | Ⅰ | LumB | Yes | Female | 61 | YES | 2 | 1 | 1 | IIA | 30 | Positive | 0 | Positive | 2+ | Positive | 30 | YES | Chemo+Tar | non-pCR |
| ZHYJ | Ⅰ | LumB | Yes | Female | 46 | NO | 2 | 2 | 0 | IIA | 90 | Positive | 55 | Positive | 2+ | Positive | 10 | YES | Chemo+Tar | non-pCR |
| GAJL | Ⅱ | LumB | Yes | Female | 40 | NO | 2 | 2 | 0 | IIA | 72 | Positive | 70 | Positive | 3+ | Unknown | 40 | YES | Chemo+Tar | non-pCR |

**Table S2.** **MUKDEN Validation Cohort information**

| **Patient Code** | **MUKDEN_ subtype** | **PAM50** | **RNA_seq** | **Sex** | **Age** | **Menopause** | **Grade** | **pT** | **pN** | **Clinical Stage** | **ER_**  **status** | **ER_**  **percent** | **PR_**  **status** | **PR_**  **percent** | **HER2_IHC Status** | **HER2_FISH Status** | **Ki67** | **Neoadjuvant Therapy** | **Therapy_ Arm** | **pCR** |
| --- | --- | --- | --- | --- | --- | --- | --- | --- | --- | --- | --- | --- | --- | --- | --- | --- | --- | --- | --- | --- |
| CYH | Ⅲ | Her2 | Yes | Female | 58 | Yes | 2 | 3 | 1 | ⅢA | Positive | 30 | Negative | 5 | 3+ | Unknown | 70 | Yes | ADC+TKI | pCR |
| YJR | Ⅰ | Her2 | Yes | Female | 47 | No | 2 | 3 | 1 | ⅢA | Positive | 30 | Positive | 40 | 3+ | Unknown | 30 | No | Adjuvant Therapy | Adjuvant Therapy |
| CYU | Ⅲ | Basal | Yes | Female | 58 | Yes | 2 | 3 | 1 | ⅢA | Positive | 30 | Negative | 5 | 3+ | Unknown | 70 | Yes | ADC+TKI | pCR |
| WZY | Ⅲ | Her2 | Yes | Female | 65 | Yes | 2 | 2 | 0 | ⅡA | Positive | 10 | Negative | 0 | 3+ | Unknown | 30 | Yes | Chemo+Tar | pCR |
| ZHJ | Ⅲ | Normal | Yes | Female | 37 | No | 2 | 2 | 1 | ⅡA | Positive | 90 | Positive | 90 | 2+ | Unknown | 30 | Yes | Chemo+Tar | non-pCR |
| WLP | Ⅳ | Basal | Yes | Female | 61 | Yes | 2 | 2 | 0 | ⅡA | Positive | 90 | Positive | 20 | 2+ | Positive | 10 | Yes | ET+CDKi+Tar | non-pCR |
| BCW | Ⅰ | LumB | Yes | Female | 60 | Yes | 2 | 3 | 1 | ⅢA | Positive | 95 | Positive | 90 | 2+ | Unknown | 40 | Yes | Chemo+Tar | non-pCR |
| LYF | Ⅲ | Her2 | Yes | Female | 54 | Yes | 3 | 2 | 1 | ⅡA | Negative | 0 | Positive | 10 | 2+ | Unknown | 60 | Yes | ADC+TKI | pCR |
| YGZ | Ⅳ | Her2 | Yes | Female | 57 | Yes | 2 | 3 | 2 | ⅢA | Positive | 5 | Positive | 20 | 3+ | Unknown | 30 | Yes | ADC+TKI | non-pCR |
| WLL | Ⅳ | LumB | Yes | Female | 43 | No | 2 | 2 | 0 | ⅡA | Positive | 80 | Positive | 20 | 2+ | Unknown | 20 | Yes | Chemo+Tar | pCR |
| YJJ | Ⅳ | Normal | Yes | Female | 54 | Yes | 2 | 2 | 0 | ⅡA | Negative | 0 | Positive | 10 | 3+ | Unknown | 15 | No | Adjuvant Therapy | Adjuvant Therapy |
| YJI | Ⅰ | Her2 | Yes | Female | 54 | Yes | 2 | 2 | 2 | ⅢA | Negative | 0 | Positive | 10 | 3+ | Unknown | 70 | Yes | Chemo+Tar | pCR |
| SGY | Ⅲ | Basal | Yes | Female | 54 | Yes | 2 | 3 | 0 | ⅡB | Negative | 0 | Positive | 10 | 3+ | Unknown | 80 | Yes | Chemo+Tar | pCR |
| SGI | Ⅳ | Normal | Yes | Female | 59 | Yes | 2 | 2 | 0 | ⅡA | Positive | 90 | Negative | 0 | 2+ | Unknown | 30 | Yes | Chemo+Tar | pCR |
| QXH | Ⅲ | Her2 | Yes | Female | 64 | Yes | 2 | 2 | 1 | ⅡA | Negative | 0 | Positive | 10 | 2+ | Unknown | 35 | Yes | Chemo+Tar | non-pCR |
| LCF | Ⅰ | Normal | Yes | Female | 52 | Yes | 2 | 2 | 0 | ⅡA | Positive | 60 | Positive | 60 | 2+ | Unknown | 70 | Yes | ET+CDKi+Tar | non-pCR |
| WXI | Ⅲ | LumB | Yes | Female | 60 | Yes | 2 | 2 | 0 | ⅡA | Positive | 95 | Positive | 70 | 2+ | Unknown | 30 | Yes | ET+CDKi+Tar | non-pCR |
| HFX | Ⅲ | Her2 | Yes | Female | 63 | Yes | 2 | 2 | 1 | ⅡA | Positive | 40 | Negative | 0 | 3+ | Unknown | 40 | No | Adjuvant Therapy | Adjuvant Therapy |
| ZYF | Ⅳ | Normal | Yes | Female | 48 | No | 2 | 2 | 1 | ⅡA | Positive | 80 | Negative | 0 | 2+ | Positive | 30 | Yes | ET+CDKi+Tar | non-pCR |
| WJL | Ⅱ | LumA | Yes | Female | 63 | Yes | 2 | 1 | 0 | ⅠA | Positive | 95 | Negative | 5 | 2+ | Unknown | 15 | No | Adjuvant Therapy | Adjuvant Therapy |
| LYY | Ⅲ | Normal | Yes | Female | 50 | Yes | 2 | 1 | 0 | ⅠA | Positive | 70 | Negative | 5 | 3+ | Unknown | 40 | No | Adjuvant Therapy | Adjuvant Therapy |
| **Patient Code** | **MUKDEN_ subtype** | **PAM50** | **RNA_seq** | **Sex** | **Age** | **Menopause** | **Grade** | **pT** | **pN** | **Clinical Stage** | **ER_**  **status** | **ER_**  **percent** | **PR_**  **status** | **PR_**  **percent** | **HER2_IHC Status** | **HER2_FISH Status** | **Ki67** | **Neoadjuvant Therapy** | **Therapy_ Arm** | **pCR** |
| LGL | Ⅲ | LumB | Yes | Female | 56 | Yes | 2 | 2 | 0 | ⅡA | Positive | 95 | Positive | 70 | 3+ | Unknown | 50 | Yes | Chemo+Tar | non-pCR |
| WYQ | Ⅱ | LumB | Yes | Female | 61 | Yes | 2 | 2 | 0 | ⅡA | Positive | 95 | Positive | 90 | 2+ | Unknown | 10 | Yes | Chemo+Tar | non-pCR |
| DIW | Ⅰ | Her2 | Yes | Female | 45 | No | 2 | 2 | 3 | ⅢC | Positive | 15 | Positive | 60 | 3+ | Unknown | 45 | Yes | ET+CDKi+Tar | non-pCR |
| XFX | Ⅳ | Normal | Yes | Female | 55 | Yes | 2 | 2 | 0 | ⅡA | Positive | 90 | Positive | 60 | 3+ | Unknown | 30 | Yes | Chemo+Tar | non-pCR |
| LFY | Ⅰ | LumA | Yes | Female | 54 | Yes | 2 | 1 | 1 | ⅡA | Positive | 95 | Positive | 20 | 3+ | Unknown | 30 | Yes | Chemo+Tar | non-pCR |
| LXY | Ⅲ | Normal | Yes | Female | 43 | No | 2 | 2 | 0 | ⅡA | Positive | 30 | Negative | 5 | 3+ | Unknown | 50 | Yes | Chemo+Tar | non-pCR |
| LSP | Ⅳ | LumA | Yes | Female | 74 | Yes | 2 | 2 | 1 | ⅡA | Positive | 70 | Positive | 80 | 2+ | Unknown | 40 | Yes | Chemo+Tar | non-pCR |
| AND | Ⅰ | Normal | Yes | Female | 31 | No | 2 | 2 | 0 | ⅡA | Positive | 90 | Positive | 90 | 3+ | Unknown | 60 | No | Adjuvant Therapy | Adjuvant Therapy |
| CQQ | Ⅱ | LumA | Yes | Female | 26 | No | 2 | 2 | 0 | ⅡA | Positive | 90 | Positive | 90 | 2+ | Unknown | 20 | No | Adjuvant Therapy | Adjuvant Therapy |
| FSF | Ⅳ | Her2 | Yes | Female | 45 | No | 2 | 2 | 0 | ⅡA | Positive | 20 | Positive | 30 | 3+ | Unknown | 20 | No | Adjuvant Therapy | Adjuvant Therapy |
| FQY | Ⅲ | Her2 | Yes | Female | 63 | Yes | 2 | 2 | 0 | ⅡA | Positive | 10 | Negative | 5 | 3+ | Unknown | 30 | No | Adjuvant Therapy | Adjuvant Therapy |
| GCA | Ⅱ | LumB | Yes | Female | 70 | Yes | 2 | 2 | 1 | ⅡA | Positive | 90 | Positive | 40 | 2+ | Positive | 70 | No | Adjuvant Therapy | Adjuvant Therapy |
| GSZ | Ⅲ | Basal | Yes | Female | 61 | Yes | 2 | 1 | 0 | ⅠA | Positive | 70 | Negative | 5 | 3+ | Unknown | 60 | No | Adjuvant Therapy | Adjuvant Therapy |
| HSH | Ⅲ | Normal | Yes | Female | 68 | Yes | 2 | 1 | 0 | ⅠA | Positive | 90 | Positive | 90 | 2+ | Unknown | 35 | No | Adjuvant Therapy | Adjuvant Therapy |
| LHW | Ⅲ | Basal | Yes | Female | 50 | Yes | 2 | 2 | 0 | ⅡA | Positive | 90 | Negative | 5 | 3+ | Unknown | 20 | Yes | ADC+TKI | non-pCR |
| ZSW | Ⅱ | LumB | Yes | Female | 58 | Yes | 2 | 2 | 1 | ⅡA | Positive | 95 | Positive | 80 | 2+ | Unknown | 40 | Yes | Chemo+Tar | non-pCR |
| CSH | Ⅱ | Her2 | Yes | Female | 52 | Yes | 2 | 2 | 0 | ⅡA | Positive | 20 | Negative | 0 | 3+ | Unknown | 15 | Yes | Chemo+Tar | pCR |
| GCC | Ⅳ | Basal | Yes | Female | 54 | Yes | 2 | 2 | 1 | ⅡA | Positive | 95 | Positive | 95 | 3+ | Unknown | 25 | Yes | ET+CDKi+Tar | pCR |
| HYH | Ⅱ | LumB | Yes | Female | 66 | Yes | 2 | 1 | 0 | ⅠA | Positive | 90 | Negative | 5 | 3+ | Unknown | 25 | No | Adjuvant Therapy | Adjuvant Therapy |
| JIY | Ⅲ | LumA | Yes | Female | 52 | Yes | 2 | 2 | 0 | ⅡA | Positive | 80 | Positive | 60 | 3+ | Unknown | 30 | No | Adjuvant Therapy | Adjuvant Therapy |
| JSH | Ⅰ | Her2 | Yes | Female | 49 | No | 2 | 2 | 0 | ⅡA | Positive | 70 | Positive | 10 | 3+ | Unknown | 60 | No | Adjuvant Therapy | Adjuvant Therapy |
| LFU | Ⅳ | Normal | Yes | Female | 51 | Yes | 2 | 2 | 0 | ⅡA | Positive | 80 | Positive | 60 | 3+ | Unknown | 15 | No | Adjuvant Therapy | Adjuvant Therapy |
| **Patient Code** | **MUKDEN_ subtype** | **PAM50** | **RNA_seq** | **Sex** | **Age** | **Menopause** | **Grade** | **pT** | **pN** | **Clinical Stage** | **ER_**  **status** | **ER_**  **percent** | **PR_**  **status** | **PR_**  **percent** | **HER2_IHC Status** | **HER2_FISH Status** | **Ki67** | **Neoadjuvant Therapy** | **Therapy_ Arm** | **pCR** |
| LHL | Ⅲ | LumB | Yes | Female | 50 | Yes | 2 | 1 | 0 | Ⅰ A | Positive | 95 | Negative | 5 | 3+ | Unknown | 60 | No | Adjuvant Therapy | Adjuvant Therapy |
| LMZ | Ⅲ | LumB | Yes | Female | 67 | Yes | 2 | 1 | 0 | ⅠA | Positive | 98 | Negative | 5 | 2+ | Unknown | 65 | No | Adjuvant Therapy | Adjuvant Therapy |
| LGF | Ⅱ | LumA | Yes | Female | 74 | Yes | 2 | 1 | 0 | ⅠA | Positive | 90 | Positive | 10 | 3+ | Unknown | 20 | No | Adjuvant Therapy | Adjuvant Therapy |
| MLF | Ⅰ | Her2 | Yes | Female | 56 | Yes | 2 | 1 | 0 | ⅠA | Positive | 90 | Negative | 0 | 3+ | Unknown | 50 | No | Adjuvant Therapy | Adjuvant Therapy |
| NJH | Ⅲ | Basal | Yes | Female | 52 | Yes | 2 | 1 | 1 | ⅡA | Positive | 10 | Negative | 0 | 3+ | Unknown | 50 | No | Adjuvant Therapy | Adjuvant Therapy |
| RFX | Ⅳ | LumA | Yes | Female | 71 | Yes | 2 | 1 | 0 | ⅠA | Positive | 95 | Positive | 90 | 2+ | Positive | 15 | No | Adjuvant Therapy | Adjuvant Therapy |
| SML | Ⅲ | LumB | Yes | Female | 48 | No | 2 | 2 | 0 | ⅡA | Positive | 90 | Positive | 90 | 3+ | Unknown | 20 | Yes | ET+CDKi+Tar | pCR |
| CJY | Ⅲ | LumB | Yes | Female | 57 | Yes | 2 | 3 | 1 | ⅢA | Positive | 95 | Positive | 70 | 2+ | Positive | 30 | Yes | ET+CDKi+Tar | non-pCR |
| GUX | Ⅱ | LumB | Yes | Female | 39 | No | 2 | 2 | 0 | ⅡA | Positive | 20 | Positive | 80 | 2+ | Unknown | 50 | Yes | Chemo+Tar | non-pCR |
| SYH | Ⅳ | Her2 | Yes | Female | 46 | No | 2 | 1 | 1 | ⅡA | Positive | 60 | Positive | 60 | 3+ | Unknown | 30 | Yes | ET+CDKi+Tar | pCR |
| YZJ | Ⅱ | LumB | Yes | Female | 54 | Yes | 2 | 2 | 0 | ⅡA | Positive | 90 | Negative | 5 | 2+ | Unknown | 30 | Yes | Chemo+Tar | non-pCR |
| SZY | Ⅱ | LumB | Yes | Female | 64 | Yes | 2 | 1 | 0 | ⅠA | Positive | 90 | Negative | 0 | 2+ | Unknown | 20 | No | Adjuvant Therapy | Adjuvant Therapy |
| WXW | Ⅳ | LumA | Yes | Female | 36 | No | 2 | 1 | 0 | ⅠA | Positive | 90 | Positive | 80 | 2+ | Positive | 40 | No | Adjuvant Therapy | Adjuvant Therapy |
| WUS | Ⅳ | Normal | Yes | Female | 38 | No | 2 | 2 | 0 | ⅡA | Positive | 90 | Positive | 90 | 2+ | Unknown | 40 | No | Adjuvant Therapy | Adjuvant Therapy |
| YAB | Ⅱ | LumB | Yes | Female | 57 | Yes | 2 | 2 | 0 | ⅡA | Positive | 95 | Positive | 70 | 2+ | Unknown | 20 | No | Adjuvant Therapy | Adjuvant Therapy |
| ZCH | Ⅰ | Her2 | Yes | Female | 51 | Yes | 2 | 2 | 0 | ⅡA | Positive | 90 | Negative | 5 | 3+ | Unknown | 40 | Yes | Chemo+Tar | non-pCR |
| LDD | Ⅲ | LumB | Yes | Female | 48 | No | 2 | 2 | 0 | ⅡA | Positive | 90 | Positive | 90 | 2+ | Unknown | 20 | Yes | Chemo+Tar | non-pCR |
| XUJ | Ⅲ | LumA | Yes | Female | 45 | No | 2 | 2 | 0 | ⅡA | Positive | 90 | Negative | 0 | 2+ | Unknown | 5 | Yes | ET+CDKi+Tar | non-pCR |
| CSX | Ⅰ | LumB | Yes | Female | 59 | Yes | 2 | 2 | 0 | ⅡA | Positive | 80 | Positive | 80 | 3+ | Unknown | 70 | Yes | ET+CDKi+Tar | non-pCR |
| CHL | Ⅰ | Her2 | Yes | Female | 60 | Yes | 2 | 2 | 1 | ⅡA | Positive | 10 | Positive | 30 | 3+ | Unknown | 50 | Yes | Chemo+Tar | pCR |
| WXH | Ⅳ | Normal | Yes | Female | 49 | No | 2 | 2 | 1 | ⅡA | Positive | 60 | Positive | 50 | 2+ | Unknown | 20 | Yes | Chemo+Tar | pCR |
| XHL | Ⅲ | Basal | Yes | Female | 33 | No | 2 | 2 | 0 | ⅡA | Positive | 95 | Positive | 95 | 3+ | Unknown | 40 | Yes | Chemo+Tar | pCR |
| **Patient Code** | **MUKDEN_ subtype** | **PAM50** | **RNA_seq** | **Sex** | **Age** | **Menopause** | **Grade** | **pT** | **pN** | **Clinical Stage** | **ER_**  **status** | **ER_**  **percent** | **PR_**  **status** | **PR_**  **percent** | **HER2_IHC Status** | **HER2_FISH Status** | **Ki67** | **Neoadjuvant Therapy** | **Therapy_ Arm** | **pCR** |
| LYL | Ⅱ | LumA | Yes | Female | 55 | Yes | 2 | 2 | 0 | ⅡA | Positive | 90 | Positive | 80 | 2+ | Positive | 20 | No | Adjuvant Therapy | Adjuvant Therapy |
| CLS | Ⅱ | LumB | Yes | Female | 42 | No | 2 | 2 | 0 | ⅡA | Positive | 85 | Positive | 50 | 3+ | Unknown | 50 | Yes | Chemo+Tar | non-pCR |
| RFZ | Ⅰ | LumB | Yes | Female | 72 | Yes | 2 | 2 | 1 | ⅡA | Positive | 95 | Negative | 5 | 3+ | Unknown | 50 | Yes | Chemo+Tar | pCR |
| ZHM | Ⅳ | Normal | Yes | Female | 37 | No | 2 | 1 | 0 | ⅠA | Positive | 20 | Negative | 2 | 3+ | Unknown | 40 | No | Adjuvant Therapy | Adjuvant Therapy |
| SHY | Ⅱ | Normal | Yes | Female | 49 | No | 2 | 2 | 0 | ⅡA | Positive | 30 | Positive | 10 | 2+ | Unknown | 40 | Yes | ET+CDKi+Tar | non-pCR |
| GYJ | Ⅱ | LumB | Yes | Female | 42 | No | 2 | 2 | 3 | ⅢC | Positive | 90 | Positive | 30 | 2+ | Unknown | 40 | Yes | ADC+TKI | non-pCR |
| FLY | Ⅱ | Basal | Yes | Female | 46 | No | 2 | 2 | 1 | ⅡA | Positive | 90 | Positive | 90 | 2+ | Unknown | 40 | Yes | ET+CDKi+Tar | pCR |
| DGJ | Ⅲ | LumA | Yes | Female | 74 | Yes | 2 | 2 | 0 | ⅡA | Positive | 90 | Positive | 90 | 3+ | Unknown | 25 | No | Adjuvant Therapy | Adjuvant Therapy |
| YHJ | Ⅳ | Basal | Yes | Female | 53 | Yes | 2 | 2 | 0 | ⅡA | Positive | 70 | Negative | 5 | 2+ | Unknown | 30 | No | Adjuvant Therapy | Adjuvant Therapy |
| ZYM | Ⅲ | Normal | Yes | Female | 56 | Yes | 2 | 2 | 1 | ⅡA | Positive | 95 | Positive | 95 | 2+ | Unknown | 30 | No | Adjuvant Therapy | Adjuvant Therapy |
| ZYY | Ⅲ | Basal | Yes | Female | 53 | Yes | 2 | 1 | 0 | ⅠA | Positive | 90 | Positive | 90 | 2+ | Unknown | 70 | No | Adjuvant Therapy | Adjuvant Therapy |
| ZYZ | Ⅱ | LumA | Yes | Female | 84 | Yes | 2 | 2 | 2 | ⅢA | Positive | 95 | Positive | 80 | 2+ | Unknown | 10 | No | Adjuvant Therapy | Adjuvant Therapy |
| ZLJ | Ⅳ | LumA | Yes | Female | 44 | No | 2 | 2 | 0 | ⅡA | Positive | 90 | Positive | 50 | 3+ | Unknown | 30 | No | Adjuvant Therapy | Adjuvant Therapy |
| HSY | Ⅱ | LumB | Yes | Female | 46 | No | 2 | 1 | 0 | ⅠA | Positive | 80 | Positive | 10 | 3+ | Unknown | 30 | No | Adjuvant Therapy | Adjuvant Therapy |
| YLY | Ⅰ | Her2 | Yes | Female | 53 | Yes | 3 | 1 | 0 | ⅠA | Positive | 60 | Negative | 5 | 3+ | Unknown | 40 | No | Adjuvant Therapy | Adjuvant Therapy |

**Table S3. List of MUKDEN-specific features in transcriptomics data-based model for MUKDEN subtype classification.**

| **MUKDEN Ⅰ (HER2-enriched)**  **Genes (70)** | **MUKDEN Ⅱ (ER-activated)**  **Genes (95)** | **MUKDEN Ⅲ (Immunomodulatory)**  **Genes (388)** | **MUKDEN Ⅳ (highly heterogeneous)**  **Genes (520)** |
| --- | --- | --- | --- |
| ASP14,CLCA2,GLYATL2,SCGB2A2,CRISP3,FGB,S100A7, CXCL17,S100A8,CEACAM6,MAB21L4,CP,S100A9,SCGB2A1,GSDMC,SPTSSB,FGG,MMP13,VTCN1,CRABP1,SOX11,AQP5,SLC34A2,AKR1B10,SDR16C5,COL11A1,GALNT3,CA9,TFAP2B,B3GNT3,DKK1,VSTM2L,EGLN3,S100A14,B4GALNT2,EPYC,GRB7,FUT3,ASRGL1,ERBB2,S100P,PROM1,KCNK1,MMP1,SCIN,PSAT1,CD24,PADI2,CCL8,TMEM45A,SLPI,POF1B,C2CD4A,MPZL2,TINCR,RSAD2,LRRC31,CXADR,PFKP,ITGB6,NRK,PGAP3,PSCA,KRT7,MED1,FGFR4,STARD3,PLEKHS1,EHF,COL10A1 | NKAIN1,RIMS4,SERPINA5,BMPR1B,PLPPR3,GFRA1,KCNJ3,KCNK15,IL20,SERPINA11,AGR3,CACNG4,ESR1,TNNT1,WNK4,ANXA9,HPN,PDZK1,PCP2,RAMP1,NEURL1,MSMB,WFDC2,AARD,FGFR3,HSPB1,SEMA3B,CHADPPM1J,MPP2,BAIAP3,SUSD3,DOK7,CCDC74A,FSIP1,CCNP,COX6C,METRN,RAPGEFL1,CCDC74B,SLC30A8,LRRC56,METTL27,SLC9A3R1,CRABP2,TMPRSS6,INSM1,ELOVL2,TUBA3D,TMC4,PLIN5,KCNF1,SLC39A6,KIF12,GREB1,PIFO,PGLYRP2,SMIM22,IGFALS,RARA,KLHDC9,STMND1,IGF1R,IGFBP2,ENHO,CACNG1,H2AJ,HDAC11,CLSTN2,SCNN1G,GATA3,C6orf141,DNALI1,ADCY1,LRRC73, NAT1,CCND1,NBPF4,ZMYND10,CGN,GAMT,KRT19,NECAB3,PIERCE1,ZNF703,PARD6B,RBM24,VSTM2A,RND2,SLC27A2,GDF15,PRR36,SMIM1,EBF4,SCNN1A | PLA2G2D,CD79A,CXCL9,CCL18,MS4A1,LYZ,MZB1,CXCL13,CD27,SLAMF7,LTB,GZMK,TNFRSF17,CD19,POU2AF1,IRF4,LGALS2,ADAMDEC1,NKG7,CCR7,SLAMF6,LAX1,ENSG00000277856,CCR2,GZMB,SIT1,ENSG00000275063,CXCR3,CD38,CD5,CD3E,LCK,CD2,S1PR4,GZMM,CTSW,SPOCK2,IL7R,PDCD1,CD6,TIGIT,CD52,CD8A,HSD11B1,CD7,KCNA3,VPREB3,PRF1,GBP5,TRAT1,SELL,CD3D,ZAP70,CST7,TBC1D10C,P2RY10,SIRPG,CCL5,PTPRC,SLAMF8,XCL2,IL2RB,PTPN7,FCRLA,FCRL5,GZMA,KLRB1,IGLL5,ICOS,ACAP1,EOMES,IL21R,IDO1,CXCR6,HLADQA2,PIM2,CD96,FYB1,SASH3,SH2D1A,IRF8,PNOC,TCL1A,GPR171,SLAMF1,CD48,CD3G,ITK,SLA2,CCR4,C16orf54,SEL1L3,GPR174,CYTIP,JSRP1,BIRC3,ADA2,CTLA4,DOK2,FCRL3,GNLY,MAP4K1,PPP1R16B,GZMH,IFNG,TBX21,TLR8,IL2RG,CD8B,SEPTIN1,GPR183,LAMP3,CYBB,CXCR5,LTA,CD247,RAC2,IKZF3,MMP9,CTSS,CORO1A,AOAH,LCP1,PLEK,IKZF1,HCST,CCR5, IL10RA,DENND1C,ZBED2,CD79B,IGSF6,JAK3,PRKCB,PYHIN1,EVI2B,CD69,CD37,SPIB,THEMIS,JAML,MRC1,TMC8,BLK,NLRC3,UBASH3A,TLR10, | SFRP1,FABP4,KRT14,ADH1B,GABRP,C7,OGN,CHRDL1,KRT5,FCER1A,ECRG4,SAA1,FMO2,CAPN6,COL14A1,ABCA8,HSPB6,SCARA5,SOX10,CAVIN2,KRT15,MEOX2,ADIPOQ,CIDEC,PLIN1,MYH11,PLIN4,COL17A1,IRX1,KRT17,ID4,LEP,RERGL,KLK5,GPD1,IGF1,MFAP4,CMA1,NGFR,MMRN1,CTSG,PTN,PI15,MEOX1,GPIHBP1,STAC2,TSPAN7,SPARCL1,CRYAB,AQP1,S100B,TIMP4,TGFBR3,TRARG1,APOD,CD300LG,CRISPLD1,INMT,TAT,NTRK2,ADH1C,EMCN,CDO1,PDK4,SYNM,STEAP4,ADAM33,ELN,FHL1, PLAC9,HBB,BTNL9,CX3CR1,LPL,DSC3,CLDN5,PRELP,CADM3,KRT6B,AOC3,THBS4,UGT2B11,RBP7,MAMDC2,FGF7,KLK7,AQP7,FGF2,CAV1, DSG3,VEGFD,CD36,PDGFD,PGM5,CIDEA,CCN3,CXCL12,ATP1A2,LTF,SOSTDC1,TNN,RBP4,TACR1,DEFB1,WIF1,IGF2,LEPR,TM4SF18,SRPX,OSR1,FAM107A,CYYR1,SERPINB5,CALML3,KIT,ITGA7,TP63,SCN4B,PDE2A,CASQ2,MATN2,CYTL1,EDNRB,HBA2,TMEM100,JAM2,ITIH5,SCGB3A1,ABCA10,SEMA3G,ABCB1,BOC,TRIM29,PLSCR4,CYBRD1,MMP7,G0S2,ANGPTL1, |

| **MUKDEN Ⅰ (HER2-enriched)**  **Genes (70)** | **MUKDEN Ⅱ (ER-activated)**  **Genes (95)** | **MUKDEN Ⅲ (Immunomodulatory)**  **Genes (388)** | **MUKDEN Ⅳ (highly heterogeneous)**  **Genes (520)** |
| --- | --- | --- | --- |
|  |  | TMEM156,CD53,CALHM6,CHIT1,CSF2RB,ITGAL,MARCO,PLA2G7,CPNE5,NCR3,RASAL3,DERL3,SNX20,EBI3,BIN2,ADGRG5,CR2,TRAF3IP3,HLADOA,BCL2A1,SPN,KLHL6,XCL1,PSTPIP1,FASLG,GPR18,ARHGAP9,MPEG1,TRAF1,UBD,CRTAM,BTLA,DPEP1,HLADRB5,GBP4,NCKAP1L,CD4,ZNF683,IL12RB1,GP1BA,BANK1,ARHGAP30,SP140,WAS,CD28,IL16,CARMIL2,TNFRSF1B,CD84,FGL2,FCRL1,PARP15,GPR132,LY9,C1QB,SCIMP,NUGGC,FOXP3,ITGB2,BCL11B,TNFRSF13C,C11orf21,WDFY4,SH2D2A,STAP1,ARHGAP45,HCLS1,NCF1,CCL17,PLAC8,CCDC69,EVI2A,HLADPB1,HLADQB2,CIITA,MMP12,CCL4,CD300LF,RASGRP2,RCSD1,BHLHA15,RIPOR2,ARHGAP25,APOBEC3G,ITGB7,PRKCQ,LILRB4,PDE6G,TESPA1,DOCK2,CTSC,STAT4,VAV1,FAM78A,PIK3CG,PTPN22,CARD11,P2RX1,PLA1A,IL18,HLADRA,FCRL2,CMKLR1,LIMD2,TFEC,P2RY13,RUNX3,TMIGD2,TNFSF14,TAGAP,HLADMB,GIMAP6,IL2RA,SELPLG,HLADPA1,IRAG2,MYO1F,TOX,HLADQB1,GCSAM,HLADOB,PARVG,MEI1,LILRA4,MYO1G,ARHGAP15,ZBP1,LPXN,ACY3,INPP5D,IL18RAP,SNAI3,FGR,ADGRE5,PD | AKAP12,PROS1,CLDN11,LYVE1,LRRN4CL,ACTG2,TNXB,RAI2,SPRY2,MAP1LC3C,DNASE1L3,PCDH18,IGFBP6,FGF10,LHFPL6,MYBPC1,SFRP4,LAMA3,FAM13C,S100A1,GPX3,MT1M,LRRC17,SVEP1,ARHGAP40,FOXC1, SLC28A3,IGSF10,HOXA5,GREM2,NPR1,MGP,C16orf89,LIFR,ZNF521,CPXM2,PCOLCE2,KRT16,LMOD1,HSPB7,AGTR1,RHOJ,SOD3,RGS5,FAM110D,ABCA9,MAOA,SORBS1,TMEM132C,TSHZ2,MET,AKR1C3,IL34,GALNT15,TINAGL1,PHGR1,NNAT,PHYHD1,CA3,CFD,DES,RSPO3,CNTFR,TCEAL7,NOVA1,LAMB3,FREM1,SMOC2,RASSF10,PTGFR,FHL5,GPC3,FAM162B,HOXA7FCGBP,LRP2,IL17B,AVPR2,LGR6,HPSE2,FRZB,LAMA2,ISM1,NDRG2,AKR1C2,DCN,CACHD1,SLIT2,NR2F1,CD302,SYNPO2,CLIC6,ANXA8L1,ST8SIA6,AK5,SAMD5,PALMD,GEM,EGFR,PDE5A,CCDC80,ADRA2A,TSPAN8,CLDN8,GNG11,PEAR1,ACACB,DPT,CCDC8,TEK,GPR146,EBF1,NRN1,OMD,TNNT3,CXCL2,PM20D2,OLFM4,PODN,TGFBR2,KCTD14,ANKRD29,CNN1,PREX2,GIPC2,GDF10,ANKRD35,DOC2B,CCDC3,CXCL1,KLF15,TMEM47, |

| **MUKDEN Ⅰ (HER2-enriched)**  **Genes (70)** | **MUKDEN Ⅱ (ER-activated)**  **Genes (95)** | **MUKDEN Ⅲ (Immunomodulatory)**  **Genes (388)** | **MUKDEN Ⅳ (highly heterogeneous)**  **Genes (520)** |
| --- | --- | --- | --- |
|  |  | CD1LG2,SRGN,CLEC12A,ITM2C,GFI1,FOLR2,PIK3CD,AKNA,FCER2,LILRB1,LILRB2,CYTH4,MATK,TNFRSF9,TIFAB,HTRA4,APOBEC3C,APOC1,PAX5,FGD2,MAL,MFNG,SLCO2B1,NCF4,GIMAP4,FERMT3,RHOF,SLA,C1QA,CD74,CCL13,RASSF4,KLRC1,VNN2,PTAFR,MCOLN2,CPVL,TNFRSF4,TRPV2,FCMR,TNFSF13B,LCP2,GMFG,SAMSN1,APBB1IP,FCN1,HVCN1,RASSF2,CD72,CD163,NPL,FMNL1,FCRL6,TASL,KLRG1,UTS2,LAIR1,ANKRD44,TNFRSF13B,GBP1,CEACAM21,HLADRB1,LAG3,RAB37,TENT5C,ZC3H12D,CHI3L1,ZNF831,APOE,CD274,CD244,FUT7,RASSF5,CCR8,KCNJ10,SLC9A9,HLADMA,LAIR2,CCL22,MS4A6A,CCDC88B,LY96,TSPAN32,HLADQA1,CD180,SCML4,APOBEC3H,CLEC4E,HK3,HCK,TNFAIP8L2,DHRS9,RNASE6,BTK,ITGAX,TMEM150B,ARHGAP4,GRAP2,IL32,LST1,C1QC,PIK3R5,GPSM3,GNG2,RAB33A,WIPF1,ZBTB32 | ADGRL4,FBLN5,DIPK2B,PPP1R14A,KCNIP2,DST,PON3,PTPRB,NOSTRIN,LDB2,GSTM5,ABCA6,SLC25A27,BMX,CRIM1,KERA,FAM3D,CAV2,LAMC2,PID1,TNMD,MYLK,PAMR1,ADCYAP1R1,CD34,CLEC14A,ERG,S1PR1,SHE,MRGPRF,MAB21L1,CES1,AOX1,RASSF9,STMN2,F2RL2,ADCY4,RAMP3,KLK10,EGR1,KCTD12,PTX3,SLIT3,KLK6,CDKN1C,SELE,OLFM1,SESN3,MME,LIPE,HLF,PLP1,CXCL14,LRRN1,IL17RD,PGR,SAA2,C8orf88,TESC,PLAAT5,GALNT16,F10,FAXDC2,LRRN3,LTBP4,DIRAS3,EFNB3,GPRASP1,VSIG2,NTN4,EBF3,SYT8,FAT2,AASS,SH3BGRL2,F13A1,MMRN2,TFPI,HSD17B11, CYP7B1,MYCT1,ADGRA2,VIT,ECM2,TXNIP,EFEMP1,CCL28,HSPA12B,CX3CL1,CPA3,FAT4,DDR2,KANK3,ITGA10,SLC27A6,GGTA1,CH25H,PELI2,MSRB3,PRTFDC1,SLC22A3,SPRY1,FZD7,ITGB8,ALX4,OXTR,TRABD2B,ADM5,RSPO1,CRTAC1,RASL12,LIMS2,LOXL4,DCDC2,A2M,SELENOP,AKR1C1,LGI4,VWF,C1QTNF7,DEPP1,ARHGEF15,PRSS12,C14orf180,TNS1,CGNL1, DDIT4L,RBMS3,ADD3,ATOH8,PDE7B,PLEKHA4,GSTA1,WLS,SLC6A14, |
| **MUKDEN Ⅰ (HER2-enriched)**  **Genes (70)** | **MUKDEN Ⅱ (ER-activated)**  **Genes (95)** | **MUKDEN Ⅲ (Immunomodulatory)**  **Genes (388)** | **MUKDEN Ⅳ (highly heterogeneous)**  **Genes (520)** |
|  |  |  | SEMA6A,ZNF662,FCN2,CYP39A1,PKDCC,CDH5,ZCCHC24,SEMA6D,CLU, CNTN1,SOX15,FLRT2,PDE1A,LHX6,IRX4,SLC19A3,AKT3,JAM3,RAMP2,CLEC4F,SMIM10,ZBTB16,PDZRN3,TNS2,ANKRD65,TMEM220,SPART,ATP1B2,MOB3B,ASPN,SCARA3,ZEB1,CHL1,CPED1,MT1E,RNF150,MRAP2,LRRC4B,ENTREP1,RHEX,RGMA,CLEC3B,ITGA9,PDGFRL,CYGB,GSN,SSPN,GIMAP8,RGS2,F3,DKK2,ALDH1A1,CBLN4,RAPGEF3,SCN7A,CLEC1A,ABCG2,SH3D19,CALCRL,TRIM2,FAM171A1,KLHL13,HIGD1B,PLPP1,PLAGL1,ID1,LGALS12,TMEM59L,BHMT2,REM1,EBF2,BMP6,MGAT3,FMOD,ADGRL2,PTH1R,BICC1,NDN,FRMD3,NR3C2,GNAL,ITGB4,C3,CRYBG3,PTPRM, MYEOV,SGCE,GLI1,TWIST2,SORBS2,EHD2,ADGRF5,KCNE4,FBLN1,PLN,MTURN,CBX7,FGF1,CPE,CNRIP1,DSEL,PDGFRA,MAML2,TAMALIN,NRIP2,P3H2,CD207,SOCS2,DLC1,SETBP1,DPYSL2,FST,HOXA3,ACSM1,PCK1 |

**Table S4. Comparisons among MUKDEN I-IV.**

|  | **MUKDEN I  N (%)** | **MUKDEN II  N (%)** | **MUKDEN III  N (%)** | **MUKDEN IV  N (%)** | | ***P^a^*** |
| --- | --- | --- | --- | --- | --- | --- |
| **Number of patients** | 41 | 38 | 30 | | 22 |  |
| **Age, years** | 50.49 | 51.53 | 52.53 | | 48.64 | 0.645 |
| **Menopause** |  |  |  | |  | 0.191 |
| Yes | 24 (58.5) | 24 (63.2) | 21 (70) | | 9 (40.9) |  |
| No | 17 (41.5) | 14 (36.8) | 9 (30) | | 13 (59. 1) |  |
| **Pathological Grade** |  |  |  | |  | 0.269 |
| II | 41 (100) | 36 (94.7) | 28 (93.3) | | 22 (100) |  |
| III | 0 (0.0) | 2 (5.3) | 2 (6.7) | | 0 (0.0) |  |
| **AJCC T category** |  |  |  | |  | 0.359 |
| pT1 | 6 (14.6) | 9 (23.7) | 7(23.3) | | 5 (22.7) |  |
| pT2 | 30 (73.2) | 28 (73.7) | 22 (73.4) | | 17 (77.3) |  |
| pT3 | 5 (12.2) | 1 (2.6) | 1 (3.3) | | 0 (0.0) |  |
| **AJCC N category** |  |  |  | |  | 0.382 |
| pN0 | 15 (36.6) | 18 (47.4) | 15 (50) | | 15 (68.2) |  |
| pN1 | 18 (43.9) | 13 (34.2) | 9 (30) | | 4 (18.2) |  |
| pN2 | 8 (19.5) | 5 (13.1) | 5 (16.7) | | 3 (13.6) |  |
| pN3 | 0 (0.0) | 2 (5.3) | 1 (3.3) | | 0 (0.0) |  |
| **HER2_IHC_Status** |  |  |  | |  | 0.164 |
| 2+ (FISH+) | 18 (43.9) | 25 (65.8) | 13 (43.3) | | 10 (45.5) |  |
| 3+ | 23 (56. 1) | 13 (34.2) | 17 (56.7) | | 12 (54.5) |  |
|  | **MUKDEN I  N (%)** | **MUKDEN II  N (%)** | **MUKDEN III  N (%)** | | **MUKDEN IV  N (%)** | ***P^a^*** |
| **Ki67** | 38.17 | 36.45 | 45 | | 34.09 | 0.124 |
| **Therapy Arm** |  |  |  | |  | 0.089 |
| ADC+TKI | 8 (19.5) | 12 (31.6) | 5 (16.7) | | 2 (9. 1) |  |
| Chemo+Tar | 18(43.9) | 8 (21. 1) | 11 (36.6) | | 9 (40.9) |  |
| ET+CDKi+Tar | 10 (24.4) | 4 (10.5) | 6 (20.0) | | 3 (13.6) |  |
| Adjuvant Therapy | 5 (12.2) | 14 (36.8) | 8 (26.7) | | 8 (36.4) |  |
| **PAM50 subtype** |  |  |  | |  | 0.0001 |
| Luminal B | 8 (19.5) | 24 (63.2) | 6 (20.0) | | 1 (4.5) |  |
| HER2-enriched | 30 (73.2) | 5 (13.2) | 15 (50) | | 2 (9. 1) |  |
| Luminal A | 0 (0.0) | 4 (10.5) | 3 (10.0) | | 8 (36.4) |  |
| Basal | 3 (7.3) | 4 (10.5) | 6 (20.0) | | 4 (18.2) |  |
| Normal | 0 (0.0) | 1 (2.6) | 0 (0.0) | | 7 (31.8) |  |

a. Two-sided Fisher’s tests were performed for categorical variables and two sided Kruskal-Wallis test was

performed for continuous variables.

**Table S5. Patient derived organoids information.**

| **PDO_ID** | **Gender** | **ER (%)** | **PR (%)** | **HER2** | **Ki-67** | **MUKDEN subtype** |
| --- | --- | --- | --- | --- | --- | --- |
| 44 | Female | 95 | 70 | 3+ | 30 | Ⅰ |
| 52 | Female | 80 | 70 | 3+ | 40 | Ⅰ |
| 69 | Female | 95 | 20 | 3+ | 60 | Ⅱ |
| 66 | Female | 90 | 80 | 3+ | 70 | Ⅱ |
| 51 | Female | 90 | 70 | 2+, FISH+ | 40 | Ⅲ |
| 84 | Female | 80 | 10 | 3+ | 30 | Ⅲ |
| 98 | Female | 80 | 95 | 3+ | 40 | Ⅳ |
| 113 | Female | 30 | 40 | 3+ | 30 | Ⅳ |

ER, estrogen receptor; PR, progesterone receptor; HER2, human epidermal growth 2
